# Ropinirole Functions Through a Dopamine Receptor D2‐Independent Mechanism to Ameliorate Amyotrophic Lateral Sclerosis Phenotypes in *TARDBP* ‐Mutant iPSC‐Derived Motor Neurons

**DOI:** 10.1111/jnc.70183

**Published:** 2025-08-18

**Authors:** Hirotsugu Asano, Tetsuya Kawaguchi, Chris Kato, Satoru Morimoto, Masato Yano, Maki Minaguchi, Daisuke Yasuda, Komei Fukushima, Hideyuki Okano

**Affiliations:** ^1^ Drug Discovery Research Department K Pharma, Inc. Fujisawa‐shi Kanagawa Japan; ^2^ Keio University Regenerative Medicine Research Center Kawasaki‐shi Kanagawa Japan; ^3^ Core Research Facilities for Basic Science, Research Center for Medical Science The Jikei University School of Medicine Minato‐ku Tokyo Japan; ^4^ Department of Pharmaceutical Sciences Osaka Medical and Pharmaceutical University Takatsuki‐shi Osaka Japan

**Keywords:** aberrant RNA splicing, amyotrophic lateral sclerosis, dopamine D2 receptor, neuronal hyperexcitation, ropinirole, TARDBP

## Abstract

Amyotrophic lateral sclerosis (ALS) is a progressive neurodegenerative disease characterized by motor neuron (MN) degeneration. Ropinirole hydrochloride (ROPI), a dopamine receptor D2 (DRD2) agonist, was identified through phenotypic screening of MNs derived from patient‐derived induced pluripotent stem cells (iPSCs) as a disease model and has emerged as a promising candidate drug for ALS treatment. The ROPALS trial, a phase I/IIa trial in patients with ALS, suggested the safety and efficacy of ROPI, albeit in a small sample size. Interestingly, a DRD2 antagonist and modulator only partially mitigated the suppressive effect of ROPI on the ALS phenotype, and the detailed mechanism of ROPI activity remains unclear. Therefore, in this study, we investigated whether the therapeutic effects of ROPI in ALS are dependent on DRD2. For this purpose, we generated DRD2‐deficient iPSCs and showed that ROPI effectively reduced neuronal cell death, reactive oxygen species (ROS) production, and neuronal hyperexcitation, independently of DRD2. Further analyses revealed that ROPI corrected aberrant RNA splicing and restored the mRNA expression of mitochondrial proteins in a DRD2‐independent manner. Our findings suggest that ROPI not only functions as a canonical DRD2 agonist but also has pleiotropic DRD2‐independent effects, offering a novel avenue for treatment strategies that target multiple pathways involved in ALS pathology.

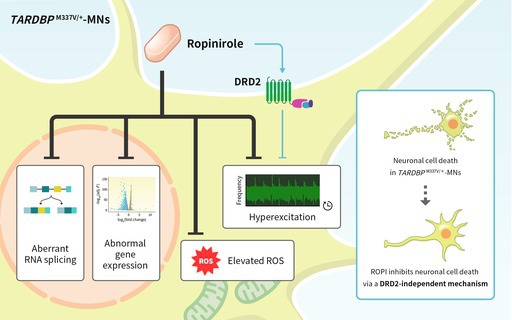

## Graphical abstract

Ropinirole, a dopamine D2 receptor (DRD2) agonist, protects motor neurons harboring ALS‐linked TARDBP mutations via both DRD2‐dependent and DRD2‐independent mechanisms. Notably, the DRD2‐independent pathway inhibits elevated reactive oxygen species (ROS), abnormal gene expression, aberrant RNA splicing, and neuronal hyperexcitation—key pathogenic features in ALS—ultimately suppressing neuronal cell death. These findings highlight a novel receptor‐independent mechanism of ropinirole that contributes to its neuroprotective effects in ALS models. This dual mechanism provides insight into ropinirole's potential therapeutic application beyond dopaminergic signaling in neurodegenerative diseases involving RNA‐binding protein dysfunction.

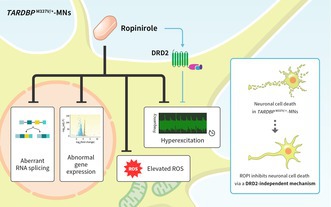

**Abbreviations**
ALS: amyotrophic lateral sclerosis
ALSFRS‐R: Amyotrophic Lateral Sclerosis Functional Rating Scale‐Revised
BDNF: brain‐derived neurotrophic factor
CTraS: chemically transitional embryoid body‐like state
DRD2: dopamine receptor D2
FUS: fused in sarcoma
GDNF: glial cell line–derived neurotrophic factor
iPSC: induced pluripotent stem cell
KBM: KBM neural stem cell medium
MN: motor neuron
PBS: phosphate‐buffered saline
RA: retinoic acid
ROPI: ropinirole hydrochloride
ROS: reactive oxygen species
TARDBP: TAR DNA‐binding protein
TDP‐43: TAR DNA‐binding protein 43

## 1 Introduction

Amyotrophic lateral sclerosis (ALS) is a neurodegenerative disease characterized by the progressive loss of motor neurons (MNs). With degeneration of both the upper and lower MNs, most patients with ALS experience muscle weakness, spasticity, muscle atrophy, and ultimately paralysis that results in death within 2–4 years (Brown and Al‐Chalabi 2017). A definitive cure has not yet been identified (Goutman et al. 2022; Okano and Morimoto 2022). Approximately 90% of ALS cases are sporadic, and only 5%–10% are familial (Mead et al. 2023). Comprehensive genetic analyses have identified numerous mutations associated with familial ALS, including mutations in the *TAR DNA‐binding protein* (*TARDBP*) gene, which encodes the TAR DNA‐binding protein 43 (TDP‐43) protein. These findings have significantly advanced our understanding of ALS pathogenesis, highlighting the role of TDP‐43 protein in disease progression (Klim et al. 2021). Both in vitro and in vivo disease model analyses have revealed diverse phenotypes, including abnormal protein aggregation, oxidative stress, mitochondrial dysfunction, and apoptosis‐related pathways (Mead et al. 2023; Cunha‐Oliveira et al. 2020). Over the past 50 years, although more than 150 therapeutic agents and strategies have been evaluated in preclinical ALS models, only riluzole, edaravone, and more recently, ultrahigh‐dose methylcobalamin have been approved in Japan. Furthermore, tofersen (an antisense oligonucleotide targeting *superoxide dismutase 1* [*SOD1*] mRNA) was approved in the USA for the treatment of ALS in adults who have a *SOD1* gene. However, these therapies show only limited benefits for ALS patients (Okano and Morimoto 2022; Oki et al. 2022; Saini and Chawla 2024). The limitations of these therapies indicate the need for novel therapeutic agents that can improve ALS of multiple etiologies.

Recently, induced pluripotent stem cell (iPSCs) have demonstrated the potential to support the development of more effective candidate drugs by recapitulating pathological processes (Okano and Yamanaka 2014; Okano and Morimoto 2022; Shi et al. 2017; Pasteuning‐Vuhman et al. 2021). In our previous research, multiphenotype screening using iPSC‐derived MNs revealed the significant potential of ropinirole hydrochloride (ROPI), a drug commonly used to treat Parkinson's disease, as a potential treatment for ALS (Fujimori et al. 2018; Okano et al. 2020, 2023). ROPI was shown to improve ALS phenotypes in patient iPSC‐derived MNs, including neurite retraction, cell death, accumulation of abnormal protein aggregates, as well as elevated levels of reactive oxygen species (ROS) and lipid peroxidation in MNs with mutations in the *TARDBP* gene, mutations in the *fused in sarcoma* (*FUS*) gene, and sporadic ALS‐MNs (Fujimori et al. 2018). Additionally, the ROPALS study, a phase I/IIa clinical trial of ROPI that included patients with ALS, demonstrated both its safety and some efficacy, as determined using the Amyotrophic Lateral Sclerosis Functional Rating Scale‐Revised (ALSFRS‐R) score (Morimoto et al. 2019, 2023). These findings suggest that better understanding the activities of ROPI could lead to improvements in ALS treatment.

ROPI was originally developed as a dopamine receptor D2 (DRD2) agonist. ROPI, along with other known DRD2 agonists such as sumanirole and bromocriptine, is thought to suppress neuronal cell death through DRD2‐mediated suppression of neuronal hyperexcitation via inhibition of cAMP production and activation of the G protein‐coupled inward rectifying channel (GIRK) (Huang et al. 2021; Morimoto et al. 2023). However, experiments with DRD2 antagonists raised the possibility that ROPI ameliorates neuronal cell death, neurite retraction, and abnormal protein aggregation in DRD2‐independent mechanisms (Fujimori et al. 2018). Therefore, the precise molecular mechanisms underlying ROPI activity remain to be elucidated.

In this study, we investigated whether the therapeutic effects of ROPI in ALS depend on DRD2. To clarify this dependency, we generated isogenic iPSCs with the *TARDBP* ^M337V/+^ mutation and a *DRD2* gene knockout. We showed that ROPI effectively reduced neuronal cell death, ROS production, and neuronal hyperexcitation, independently of DRD2. Further analyses revealed that ROPI corrected aberrant RNA splicing and restored the mRNA expression of mitochondrial proteins in a DRD2‐independent manner. These findings suggest that ROPI's multifunctional properties, including DRD2‐independent effects, may underlie its therapeutic efficacy in ALS. Furthermore, these findings could provide new insights into the underlying mechanisms of ALS treatment.

## 2 Materials and Methods

### 2.1 Culture of Undifferentiated iPSCs

Control human iPSCs (RPChiPS8023G1; REPROCELL) and isogenic iPSCs were cultured in StemFit AK02N (Ajinomoto) in an atmosphere containing 5% CO_2_ and subsequently dissociated using 0.5 × TrypLE Select (Thermo Fisher Scientific) and seeded at a density of 1.0 × 10^4^ cells/well in 6‐well plates treated with iMatrix‐511 (Takara Bio), a recombinant laminin E8 fragment that enhances cell attachment. A total of 10 μM Y‐27632 (Chemscene), a ROCK inhibitor known to suppress cell death, was added on only the first day. The culture medium was changed every other day for 1 week. iPSCs were passaged every 7 days. The iPSC line used in this study (StemRNA Human iPSC 802‐3G, Reprocell) is not listed as a commonly misidentified cell line by the International Cell Line Authentication Committee. The parental iPSC line (StemRNA Human iPSC 802‐3G, Reprocell) was used to generate both the TARDBP M337V knock‐in and DRD2 knockout (KO) lines via CRISPR/Cas9 genome editing. STR profiling was not performed after editing. However, the identity of each edited iPSC clone was confirmed by Sanger sequencing of the edited loci and karyotype analysis to ensure genomic integrity.

### 2.2 Generation of Isogenic iPSCs via CRISPR/Cas9 Genome‐Editing Technology

Using the Alt‐R CRISPR/Cas9 system (IDT), isogenic iPSCs carrying a *TARDBP M337V* heterozygous mutation (*TARDBP* ^*M337V/+*^) were generated from healthy control RPChiPS8023G1 iPSCs (*TARDBP* ^+/+^). On the day before transfection, the iPSCs (2 × 10^5^) were seeded into 6‐well plates. crRNA and tracrRNA (Alt‐R CRISPR/Cas9 crRNA and tracrRNA) were resuspended in nuclease‐free duplex buffer and hybridized for 5 min at 95°C to form a gRNA duplex. Alt‐R S.p. HiFi Cas9 Nuclease V3 is mixed with the gRNA duplex in Opti‐MEM medium to form Cas9 RNP. Next, Cas9 RNP and a single‐strand oligo donor nucleotide (ssODN) were introduced using the Lipofectamine Stem Transfection Reagent (Thermo Fisher Scientific) and Opti‐MEM (Gibco). After 24 h, the medium was changed to StemFit AK02N supplemented with 10 μM Y‐27632. After 48 h, the cells were seeded in 96‐well plates at a density of 0.5 cells/well. Single clones were maintained with StemFit AK02N, and the cells were partially lysed to prepare the PCR template. The genomic region flanking the CRISPR target site was amplified using PCR, and its sequence was confirmed via Sanger sequencing.

To generate *DRD2* knockout cell lines (*TARDBP* ^*M337V/+*^; *DRD2* ^*−/−*^), guide RNAs for the CRISPR/Cas9 system were designed near the transcription start site and inner region of the *DRD2* gene locus. Guide sequences were cloned and inserted into sgRNA‐expressing and humanized Cas9‐encoding plasmids acquired from VectorBuilder Inc. The plasmids were transfected using the Lipofectamine Stem Transfection Reagent. For the clonal selection of mutants, cells were selected in StemFit AK02N with 1.0 μg/mL blasticidin (InvivoGen) for 24 h. Single clones were checked as described above.

### 2.3 Introduction of a Polycistronic Vector Into iPSCs

Tet‐on‐inducible iPSCs were modified with NIL transcription factors using the following vectors: pPB‐Puro‐TRE3G>hNEUROG2:T2A:hISL1:T2A:hLHX3, pRP‐Puro‐CMV‐hyPBase, and pPB‐Hygro‐EF1A>Tet3G (VectorBuilder). These vectors were cotransfected into iPSCs using the Lipofectamine Stem Transfection Reagent. After 2 days of incubation, the transfected iPSCs were cultured in StemFit AK02N medium supplemented with 200 ng/mL puromycin (InvivoGen) and 150 μg/mL hygromycin B Gold (InvivoGen). The surviving colonies were propagated after a single clone was selected and then appropriately stored using StemCellBanker (ZENOAQ) until use. Tet‐on‐inducible iPSC clones used in the assays were at passages ≤ 45. Although genome editing and NIL vector introduction contributed to an increase in passage number, only clones with a normal karyotype, verified genomic integrity, and stable morphology were selected for differentiation and subsequent analyses.

### 2.4 MN Induction In Vitro

Using TrypLE Select, iPSCs were dissociated into single cells and plated on an iMatrix‐511 with StemFit AK02N and Y‐27632. On the following day, the iPSCs were stimulated to adopt a chemically transitional embryoid body‐like state (CTraS) using 3 μM SB431542 (Tocris), 150 μM LDN‐193189 (StemRD), and 3 μM CHIR99021 (Sigma–Aldrich) (Fujimori et al. 2017). After 5 days, the CTraS‐treated iPSCs were dissociated into single cells with TrypLE Select and plated on poly‐D‐lysine (Sigma–Aldrich)‐ and Matrigel (Corning)‐coated plates with MN medium, KBM neural stem cell medium (KBM; Kohjin Bio) containing 2% B27 supplement (Thermo Fisher Scientific), 10 ng/mL brain‐derived neurotrophic factor (BDNF; Abcam), 10 ng/mL glial cell line–derived neurotrophic factor (GDNF; Alomone Labs), 2 μM retinoic acid (RA; Sigma–Aldrich), 200 ng/mL ascorbic acid (Sigma–Aldrich), 2 μM DAPT (Selleck), and 2 μM PD‐0332991 (Selleck). For the first 5 days after seeding iPSCs, 1 μg/mL doxycycline was treated, followed by culture for an additional 5–14 days at 37°C in a humidified atmosphere containing 5% CO_2_. Starting on Day 5, ROPI was added to the MNs, and half of the medium was replaced every 2–3 days.

### 2.5 Sequencing Analysis

Genomic DNA was isolated using a Wizard Genomic DNA Purification Kit (Promega) from *TARDBP* ^*+/+*^‐, *TARDBP* ^*M337V/+*^‐, or *TARDBP* ^*M337V/+*^; *DRD2* ^*−/−*^‐iPSCs. Sequencing analysis was outsourced to Eurofins Genomics. Details of the primer sequences are presented in Table S1.

### 2.6 Immunocytochemistry

The cells were fixed for 20 min at room temperature in phosphate‐buffered saline (PBS) containing 4% paraformaldehyde, then blocked for 1 h in PBS with 3% BSA and 0.1% Triton X‐100, and incubated overnight at 4°C with the primary antibodies described in Table S2. The cells were then rinsed with PBS and incubated with species‐specific Alexa Fluor 488‐, Alexa Fluor 555‐, or Alexa Fluor 647‐conjugated secondary antibodies (1:1000; Thermo Fisher Scientific), followed by nuclear counterstaining with DAPI. Images were obtained using an HS All‐in‐One Fluorescence Microscope (BZ9000; Keyence) or IN Cell Analyzer 6000 (GE Healthcare).

### 2.7 RT–qPCR

Total RNA was isolated using the RNeasy Plus Mini Kit (Qiagen), and cDNA was prepared using the ReverTra Ace qPCR RT Kit (Toyobo). RT–qPCR was performed with THUNDERBIRD SYBR qPCR Mix (Toyobo) on a ViiA 7 Real‐Time PCR System (Thermo Fisher Scientific). The details of the primers used are presented in Table S1.

### 2.8 Western Blotting

Total protein was extracted with RIPA buffer supplemented with a protease inhibitor cocktail and a phosphatase inhibitor cocktail (Nacalai Tesque), and the protein concentration was determined using the Micro BCA Protein Assay Kit (Thermo Fisher Scientific). Total protein was separated by SDS–PAGE via 4%–15% Mini‐PROTEAN TGX precast gels (Bio–Rad) and then transferred to a PVDF membrane using a Trans‐Blot Turbo Transfer System (Bio–Rad). The membrane was then probed with the following primary antibodies: anti‐β‐actin (1:1000; Sigma–Aldrich) and anti‐dopamine receptor D2 (1:1000; Frontier Lab) in Blocking one (Nacalai Tesque). After incubation, the membranes were washed more than three times with TBS (Nacalai Tesque) containing 0.1% Tween‐20. Detailed information regarding the primary antibodies used is summarized in Table S2. The signals were detected with horseradish peroxidase‐conjugated secondary antibodies (Cell Signaling Technology) using an ECL Prime kit (Amersham Biosciences). Quantitative analysis was performed using a ChemiDoc XRS+ System (Bio–Rad).

### 2.9 Simple Western

Sample and antibody preparation and reagent loading were performed according to the ProteinSimple user manual. For performing Simple Western experiments on the Wes, the 12–230 kDa Wes Separation Module, containing 8 × 25 capillary cartridges, reagents, and consumables, was utilized. The primary antibodies used were anti‐CREB (1:50) and anti‐Phospho‐CREB (Ser133) (1:50), both obtained from Cell Signaling Technology. Automated separation, electrophoresis, and chemiluminescence detection were performed using the default parameters of the ProteinSimple WES instrument (ProteinSimple). The resulting digital images were analyzed using Compass software (ProteinSimple). Detailed information regarding the primary antibodies used is summarized in Table S2.

### 2.10 Cell Death Assays

MNs were cultured in MN medium for 5 days; thereafter, half of the medium was replaced with a 1:1 mixture of KBM and Neurobasal medium (Thermo Fisher Scientific) containing BDNF, GDNF, and RA. On Day 9, the MNs were cultured for 2 h with SYTOX Green Nucleic Acid Stain (1:500; Thermo Fisher Scientific), Tubulin Tracker Deep Red (1:2000; Thermo Fisher Scientific), and Hoechst 33258 (0.5 μg/mL; Sigma–Aldrich). Images were obtained using an IN Cell Analyzer 6000. Details of the probes used are presented in Table S3.

### 2.11 ROS Production Assays

MNs were cultured in MN medium for 5 days, after which the medium was replaced with antioxidant‐free medium, a 1:1 mixture of KBM and neurobasal medium supplemented with 2% B27 supplement minus antioxidants (Thermo Fisher Scientific), BDNF, GDNF, and RA. ROPI or 5‐hydroxyoxindole was added to the medium starting from 5 days. On Day 9, the MNs were cultured with CellROX Green Reagent for oxidative stress detection (Thermo Fisher Scientific) and with Hoechst 33258 for 2 h. Images were obtained using an IN Cell Analyzer 6000. The details of the probes used are presented in Table S3.

### 2.12 High‐Content Analysis

For the cell population, fluorescence intensity, cell death, and ROS production assays, stained plates were imaged using an IN Cell Analyzer 6000; a set of 4 × 4 fields was collected from each well using a 20× objective. The analysis (IN Cell Developer Toolbox v1.9; GE Healthcare) began with the identification of intact Hoechst 33258‐ or DAPI‐stained nuclei, which were defined as traced nuclei that had a surface area > 30 μm^2^. To determine the differentiation efficiency, the proportion of cells differentiating into neurons (number of β III tubulin‐positive cells/number of DAPI‐positive cells) and the proportion of MNs among neurons (number of HB9‐positive cells/number of β III tubulin‐positive cells) were quantified. For the cell death assay, the proportion of SYTOX‐positive cells (number of SYTOX‐positive cells/number of Hoechst 33258‐positive cells) was quantified. For the ROS production assays, the CellROX fluorescence intensity (total intensity of CellROX Green/Hoechst 33258‐positive cells) was quantified.

### 2.13 Microelectrode Array

The microelectrode assay (MEA) was performed using the Maestro system (Axion Biosystems). Neuronal induction of *TARDBP* ^*+/+*^‐, *TARDBP* ^*M337V/+*^‐, or *TARDBP* ^*M337V/+*^; *DRD2* ^*−/−*^‐iPSCs was performed in 48‐well MEA plates coated with 0.15% polyethyleneimine and 1% Matrigel. Each well was seeded with 2.0 × 10^5^ cells in 50 μL of culture medium. At 1 h after seeding, 450 μL of culture medium was added. In addition to neuronal induction, for other analyses, Y‐27632, doxycycline, RA, purmorphamine, and iMatrix‐511 silk were included in the medium for the first 5 days, and after Day 5, half of the medium was changed every 3–4 days. Data were acquired at a sampling rate of 12.5 kHz and filtered through a 200–3000 Hz Butterworth bandpass filter. The detection threshold was set to 6 × SD of the baseline electrode noise. Spontaneous activity was recorded for 5 min at 37°C. The number of spikes was measured using the Axion Integrated Studio (Axion Biosystems). The weighted mean firing rate was obtained by weighting each electrode's firing rate by its spike count.

### 2.14 RNA Sequencing and Analysis

Total RNA was extracted using an RNeasy Plus Mini Kit (Qiagen) according to the manufacturer's protocol. The RNA concentration was measured using a NanoDrop instrument (Thermo Fisher Scientific), and RNA quality was analyzed using an Agilent 2100 Bioanalyzer (Agilent Technologies). A cDNA library was created using the TruSeq Stranded mRNA Library Prep Kit (Illumina). The libraries were sequenced via paired‐end sequencing using a NovaSeq 6000 System (Illumina). All procedures were performed by Macrogen Inc. For quality control, raw FASTQ files were processed using fastp (version 0.23.2; ‐q 15 ‐n 5 ‐l 15 ‐w 8, with other parameters set to default values) (Chen et al. 2018). Following quality control, sequencing reads (FASTQ files) were mapped to the human GRCh38 genome (GENCODE GRCh38.p13) using HISAT2 (version 2.1.0) with default parameters (Kim et al. 2019). Exon–exon junction reads were subsequently extracted using RegTools (version 0.5.2; ‐a 6 ‐m 50 ‐M 500000 ‐s 0, with other parameters set to default values) (Cotto et al. 2023). Salmon (version 1.7.0; reference index generated using GENCODE GRCh38.p13; ‐l A‐p 8, with other parameters set to default values) was used to calculate read counts (Patro et al. 2017). Time series analysis was performed using maSigPro (version 1.66.0), and differential expression analysis was conducted using DESeq2 (version 1.34.0) (Conesa et al. 2006; Love et al. 2014). Genes with an adjusted *p* value < 0.05 and |log_2_(fold change)| > 1 were considered differentially expressed (Melamed et al. 2019; NeuroLINCS Consortium et al. 2021; Setsu et al. 2025). LeafCutter (version 0.2.9) was used for differential intron splicing analysis with all parameters set to their default values (Li et al. 2018), consistent with previous ALS studies that successfully identified mis‐splicing events (Ma et al. 2022). The uniform manifold approximation projection was performed using Umap (version 0.2.10.0). GO and KEGG pathway analyses were performed using g:profiler (“term size” option set to “5–350,” and other parameters set to default values) (Kanehisa et al. 2023; Kanehisa 2019; Kanehisa and Goto 2000; Gene Ontology Consortium 2021; Ashburner et al. 2000; Reimand et al. 2019; Raudvere et al. 2019).

### 2.15 Statistical Analysis

Prior to statistical comparisons, data normality was assessed using the Kolmogorov–Smirnov normality test. All datasets showed no significant deviation from normality (*p* > 0.05), justifying the use of parametric analyses. Statistical analyses were performed using ANOVA, followed by Tukey's test, Welch's *t*‐test, or Games–Howell test, as appropriate. The equality of variances was first assessed using the *F*‐test for two‐group comparisons and Bartlett's test for analyses involving more than two groups. If equal variances were confirmed, ANOVA followed by Tukey's test (for multiple comparisons) was used. If variances were unequal, Welch's *t*‐test was applied for two‐group comparisons, and the Games–Howell test was applied for multiple‐group comparisons. All statistical tests were two‐sided. Data are presented as means ± standard error (SE) or standard deviation (SD). Prior to statistical testing, outliers were identified and excluded using the Smirnov–Grubbs test. All statistical analyses were performed using EZR software (Kanda 2013). Statistical analyses were performed using appropriate tests as described, and detailed results are provided in Table S4. No blinding was performed during the study. All experiments, including data collection and analysis, were conducted by researchers who were aware of the group assignments.

## 3 Results

### 3.1 Genome‐Editing of *DRD2* Gene Deficient in *TARDBP* ^*M337V*^ Mutant iPSCs

**FIGURE 1:**
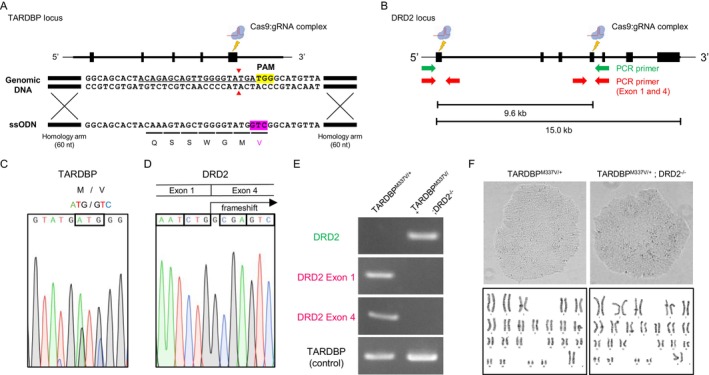
Generation of *TARDBP* ^*M337V/+*^; *DRD2* ^*−/−*^‐iPSCs. (A) Schematic diagram of the CRISPR/Cas9‐mediated *TARDBP* mutant genome‐editing protocol in iPSCs. Homologous recombination was performed using ssODNs containing homology arms flanking the M337V mutation site in the *TARDBP* region. (B) Schematic diagram of the CRISPR/Cas9‐mediated *DRD2* deletion genome‐editing protocol in iPSCs. (C) *TARDBP* ^*M337V*^ (ATG‐to‐GTC) heterozygous mutations identified in CRISPR/Cas9‐mediated iPSCs. (D) *DRD2* homozygous deletion identified in CRISPR/Cas9‐mediated iPSCs. (E) Genomic PCR analysis of the *DRD2* gene region in the *TARDBP* ^*M337V/+*^; *DRD2* ^*−/−*^ strain. (F) Representative images displaying the morphology of iPSCs (top panel) and the karyotypes (bottom panel) associated with both *TARDBP* ^*M337V/+*^ and *TARDBP* ^*M337V/+*^; *DRD2* ^*−/−*^‐iPSCs.

To clarify whether a DRD2‐independent mechanism mediates suppression of the ALS phenotype by ROPI, we generated *DRD2* gene‐deficient iPSCs to allow us to test the effects of ROPI on MNs derived from these cells. Isogenic iPSCs with a *TARDBP* ^M337V^ heterozygous mutation (*TARDBP* ^*M337V/+*^; Figure 1A) and *DRD2* knockout (*TARDBP* ^*M337V/+*^; *DRD2* ^*−/−*^; Figure 1B) were established. Sanger sequencing revealed an M‐to‐V transition in the *TARDBP* gene (Figure 1C) and a deletion spanning exons 1–4 in the *DRD2* gene (Figure 1D). The deletion mutant caused a frameshift such that the protein was translated with six amino acids from Exon 1 and an irregular 93‐amino acid downstream sequence. In addition, PCR of genomic DNA produced bands when primers outside the deletion region were used, whereas no band was detected via PCR using primers amplifying Exon 1 or Exon 4 (Figure 1E). Off‐target effects of *TARDBP* and DRD2 gRNAs were assessed by sequencing the top 10 candidate regions identified using the UCSC Genome Database, with no genome editing‐induced mutations detected (Table S5). Isogenic ALS‐iPSCs displayed a typical colony morphology that resembled that of human embryonic stem cell lines, and G‐band staining demonstrated a normal karyotype (Figure 1F).

### 3.2 MN‐Differentiation of the *TARDBP^M337V/+^*; *DRD2* ^*−/−*^‐iPSCs, and Validation of Dopamine Receptor Expression

**FIGURE 2:**
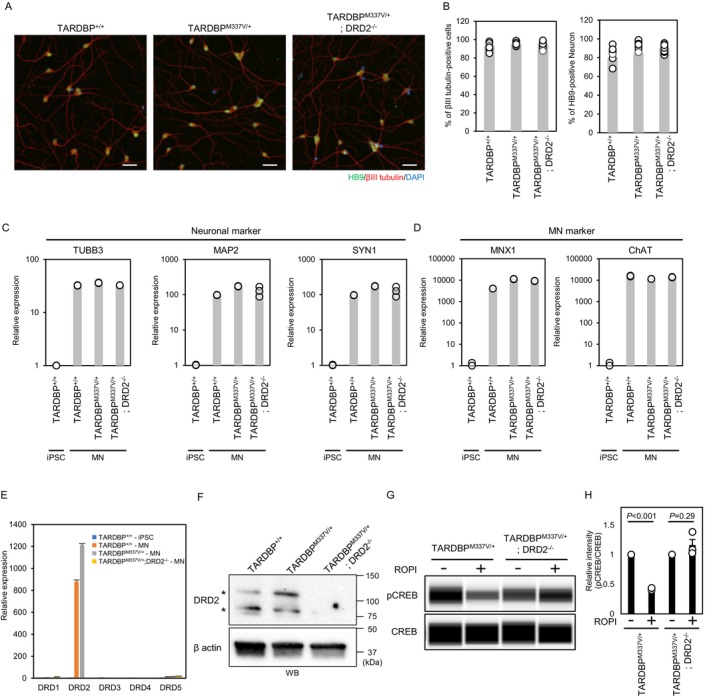
Characterization of *TARDBP* ^*M337V/+*^; *DRD2* ^*−/−*^‐MNs. (A) Representative image of immunocytochemistry for MN markers (HB9) and a neuronal marker (βIII‐tubulin). (B) Quantitative immunocytochemical analysis of neuronal and MN marker expression in *TARDBP* ^*+/+*^, *TARDBP* ^*M337V/+*^, and *TARDBP* ^*M337V/+*^; *DRD2* ^*−/−*^‐MNs. The values represent the means ± SDs. (C–E) RT–qPCR analysis of the expression of neuronal markers (*TUBB3*, *MAP2*, *SYN1*) (C), MN markers (*MNX1*, *ChAT*) (D), and DRD family genes (E) in *TARDBP* ^*+/+*^, *TARDBP* ^*M337V/+*^, and *TARDBP* ^*M337V/+*^; *DRD2* ^*−/−*^‐MNs. (F–H) Immunoblotting analysis of DRD2 protein expression (F) and CREB phosphorylation (G) in *TARDBP* ^*+/+*^, *TARDBP* ^*M337V/+*^, and *TARDBP* ^*M337V/+*^; *DRD2* ^*−/−*^‐MNs. (H) Quantitative analysis of CREB phosphorylation by immunoblotting in *TARDBP* ^*M337V/+*^ and *TARDBP* ^*M337V/+*^; *DRD2* ^*−/−*^‐MNs with or without ROPI (*n* = 3 independent experiments; mean ± SEM, Welch's *t*‐test).

First, we examined whether motor neurons could be induced from isogenic iPSCs with DRD2 deletion or TARDBP mutations, as well as from healthy control iPS cells. MNs have been previously induced from iPSCs through the overexpression of three transcription factors, referred to as NIL factors: neurogenin 2 (NGN2), ISL LIM homeobox 1 (ISL1), and LIM homeobox protein 3 (LHX3) (Imamura et al. 2017; Morimoto et al. 2023; Setsu et al. 2025). Here, we established an iPSC line carrying NIL factors using the PiggyBac transposon system, with their expression induced by doxycycline via the Tet‐On expression system (Figure S1A). For MN differentiation, iPSCs were directed into neuroepithelial cells using the CTraS method and cultured as single cells in doxycycline‐containing MN medium (Figure S1B; Fujimori et al. 2017; Imamura et al. 2017). The protein expression of MN markers was examined by immunocytochemistry using the anti‐neuronal marker βIII‐tubulin and the anti‐MN marker HB9, with the nuclear stain DAPI. The ratio of βIII‐tubulin‐positive cells to total cells exceeded 90%, and a neuronal morphology was observed (Figure 2A,B left). The percentage of HB9‐positive cells among the neurons exceeded 80% (Figure 2B, right). These data show that isogenic iPSCs exhibit high‐efficiency MN differentiation, similar to that of healthy iPSCs. Expression analysis of additional MN markers via RT–qPCR showed higher expression of the neuronal markers TUBB3, MAP2, and SYN1 compared with that in iPSCs, which served as negative controls (Figure 2C). Additionally, compared with iPSCs, the induced cells presented sufficient expression of the MN markers HB9 and ChAT (Figure 2D). These data demonstrated that *TARDBP* ^*+/+*^‐*MN*, *TARDBP* ^*M337V/+*^‐MN, and *TARDBP* ^*M337V/+*^; *DRD2* ^*−/−*^‐MNs can differentiate into MNs.

Expression analysis of dopamine receptors in MNs revealed increased levels of *DRD2* mRNA expression in both wild‐type and *TARDBP* ^*M337V/+*^ MNs, whereas no *DRD2* mRNA expression was detected in *TARDBP* ^*M337V/+*^; *DRD2* ^*−/−*^ MNs (Figure 2E, Figure S2). Further analyses demonstrated that DRD2 is the major dopamine receptor and that other dopamine receptors are expressed at barely detectable levels and were not upregulated to compensate for *DRD2* knockout in MNs. The expression of the DRD2 protein was confirmed in *TARDBP* ^*+/+*^‐MNs and *TARDBP* ^*M337V/+*^‐MNs, whereas it was undetectable in *TARDBP* ^*M337V/+*^; *DRD2* ^*−/−*^‐MNs (Figure 2F, Figure S3). Furthermore, we investigated the phosphorylation of CREB, a PKA target suppressed by the DRD2 signaling pathway. Functional assays indicated that the activation of DRD2 signaling by ROPI resulted in decreased CREB phosphorylation in *TARDBP* ^*M337V/+*^‐MNs (relative to the control: 0.42; *p* < 0.001 by paired *t*‐test, Figure 2G,H). In contrast, CREB phosphorylation was not decreased by ROPI treatment in *TARDBP* ^*M337V/+*^; *DRD2* ^*−/*−^‐MNs (relative to the control: 1.16; *p* = 0.29 by paired *t*‐test, Figure 2G,H). Therefore, these iPSCs provide a tool for the analysis of DRD2‐independent mechanisms of ROPI.

### 3.3 ROPI Inhibited Cell Death and Its Underlying Mechanism

**FIGURE 3:**
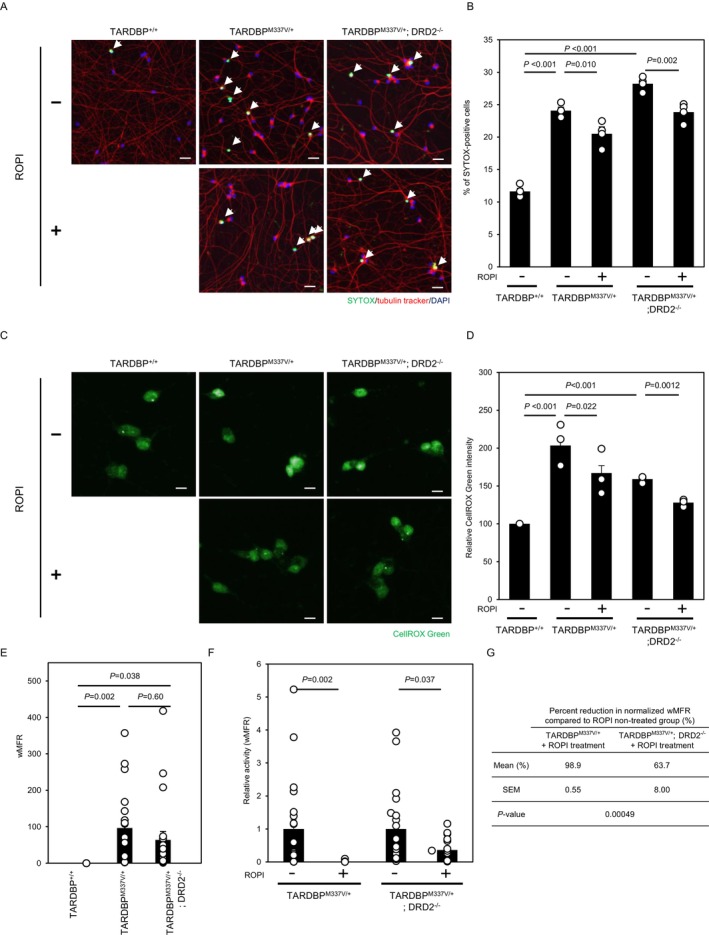
ROPI suppresses the ALS phenotype in *TARDBP* ^*M337V/+*^ and *TARDBP* ^*M337V/+*^; *DRD2* ^*−/−*^ mutant cells. (A) Representative images of in *TARDBP* ^*+/+*^, *TARDBP* ^*M337V/+*^, and *TARDBP* ^*M337V/+*^; *DRD2* ^*−/−*^‐MNs stained for the cell death probe (SYTOX Green), neurite probe (tubulin tracker), and DAPI. (B) Quantitative data of the ratio of SYTOX‐positive cells to DAPI‐positive cells (*n* = 3 independent experiments; mean ± SEM, one‐way ANOVA with Tukey's test). (C) Representative images depicting staining for the ROS detected probe (CellROX Green), neurite probe (tubulin tracker), and DAPI in *TARDBP* ^*+/+*^, *TARDBP* ^*M337V/+*^, and *TARDBP* ^*M337V/+*^; *DRD2* ^*−/−*^‐MNs. (D) Quantitative data of the average ROS intensity as a function of the number of DAPI‐stained cells (*n* = 3 independent experiments; mean ± SEM, Welch ANOVA followed by Games–Howell test). (E) The weighted mean firing rate (wMFR) on Day 16 after plating in human iPSC‐derived MNs with *TARDBP* ^*+/+*^, *TARDBP* ^*M337V/+*^, and *TARDBP* ^*M337V/+*^; *DRD2* ^*−/−*^ (*n* = 3 independent experiments [6–9 well per condition]; mean ± SEM, Welch ANOVA followed by Games–Howell test). (F) Relative wMFR activity upon addition of 10 μM ROPI in *TARDBP* ^*M337V/+*^ and *TARDBP* ^*M337V/+*^; *DRD2* ^*−/−*^‐MNs on Day 16 of culture (*n* = 3 independent experiments, 6–9 wells per condition; mean ± SEM; Welch's *t*‐test). (G) Comparison of suppression rates by ROPI in *TARDBP* ^*M337V/+*^ and *TARDBP* ^*M337V/+*^; *DRD2* ^*−/−*^‐MNs (*n* = 3 independent experiments; mean ± SEM, Welch's *t*‐test).

To assess the potential DRD2‐independent effects of ROPI, we utilized DRD2 knockout MNs. Initially, we investigated whether cell death was inhibited through a DRD2‐independent ROPI mechanism. Both *TARDBP* ^*M337V/+*^‐MNs and *TARDBP* ^*M337V/+*^; *DRD2* ^*−/−*^‐MNs exhibited a drastic increase in SYTOX‐positive cells, which indicates greater neuronal cell death than was observed in *TARDBP* ^*+/+*^‐MNs (Figure 3A,B). ROPI treatment reduced the number of SYTOX‐positive *TARDBP* ^*M337V/+*^MN cells. Remarkably, ROPI treatment effectively inhibited cell death in *TARDBP* ^*M337V/+*^; *DRD2* ^*−/*−^‐MNs. Additionally, in the absence of exogenous dopamine or D2 agonists, a slight increase in cell death was also observed in DRD2 knockout cells (Figure 3A,B, Table S4). However, the underlying mechanism remains unclear and requires further investigation. These data suggest that a DRD2‐independent mechanism is important for the ROPI‐mediated suppression of neuronal cell death.

### 3.4 ROPI Reduced ROS and Its Underlying Mechanism

Next, in light of previous evidence that ROPI effectively decreased ROS levels in MNs with the TARDBP mutation, which may be important for ALS pathology, we investigated whether ROPI plays a role in reducing ROS production via the dopamine signaling pathway. Both *TARDBP* ^*M337V/+*^‐MNs and *TARDBP* ^*M337V/+*^; *DRD2* ^*−/−*^‐MNs exhibited significantly greater ROS levels than *TARDBP* ^*+/+*^‐MNs did (Figure 3C,D). Treatment with ROPI induced a notable reduction in ROS levels in *TARDBP* ^*M337V/+*^‐MNs. Importantly, ROPI treatment effectively reduced ROS levels in *TARDBP* ^*M337V/+*^; *DRD2* ^*−/*−^‐MNs (Figure 3C,D). Additionally, we investigated the DRD2‐independent reduction in ROS using 5‐hydroxyoxindole, a ROPI analog that lacks DRD2 activity. The ROPI analog also inhibited the increase in ROS levels in *TARDBP* ^*M337V/+*^‐MNs (Figure S4). These findings suggest that ROPI functions to reduce ROS production in a DRD2‐independent manner.

### 3.5 ROPI Reduced the Hyperexcitability of *TARDBP* ^*M337V*/+^‐MNs and Its Underlying Mechanisms

In our previous study, we observed spontaneous hyperexcitability in iPSC‐MNs with ALS mutations and found that ROPI significantly inhibited this hyperexcitability (Morimoto et al. 2023; Kondo et al. 2023). We investigated whether the hyperexcitability of iPSC‐MNs was inhibited by ROPI through a DRD2‐independent mechanism. The spontaneous firing of *TARDBP* ^*M337V/+*^‐MNs and *TARDBP* ^*M337V/+*^; *DRD2* ^*−/−*^‐MNs increased starting from Day 9 (Figure S5). Compared with *TARDBP* ^+/+^‐MNs, both *TARDBP* ^*M337V/+*^‐MNs and *TARDBP* ^*M337V/+*^; *DRD2* ^*−/−*^‐MNs exhibited a dramatic increase in hyperexcitability (Figure 3E). ROPI treatment was initiated from Day 14, when spontaneous firing had stabilized. ROPI treatment inhibited the hyperexcitability of *TARDBP* ^*M337V/+*^‐MNs (Figure 3F). Remarkably, ROPI treatment also effectively suppressed hyperexcitability in *TARDBP* ^*M337V/+*^; *DRD2* ^*−/−*^‐MNs (Figure 3F).

We investigated whether there were differences in the responsiveness to ROPI induced by DRD2 knockout by examining the inhibition ratio of ROPI‐induced hyperexcitation in *TARDBP* ^*M337V/+*^‐MNs and *TARDBP* ^*M337V/+*^; *DRD2* ^*−/−*^‐MNs cells. The inhibitory effect of ROPI on *TARDBP* ^*M337V/+*^; *DRD2* ^*−/−*^‐MNs was attenuated compared with the inhibitory effect of ROPI on *TARDBP* ^*M337V/+*^‐MNs (98.9% in *TARDBP* ^*M337V/+*^‐MNs and 63.7% in *TARDBP* ^*M337V/+*^; *DRD2* ^*−/−*^‐MN; Figure 3G). These data suggest that the inhibition of hyperexcitability by ROPI involves both DRD2‐independent and DRD2‐dependent mechanisms.

### 3.6 ROPI Rescues Abnormal Gene Expression in *TARDBP* ^*M337V/+*^‐MNs and Its Underlying Mechanisms

**FIGURE 4:**
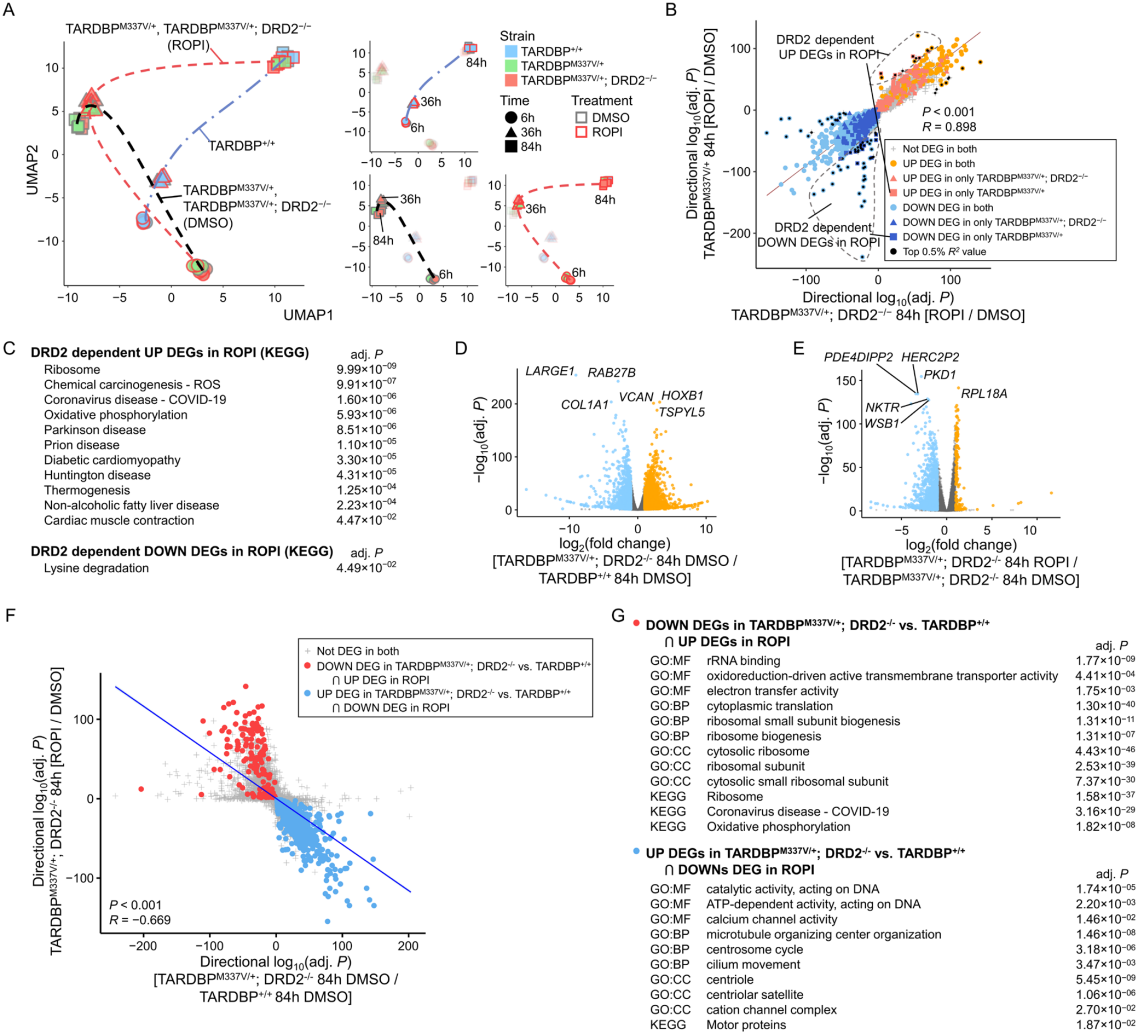
ROPI rescues abnormal gene expression caused by the *TARDBP* ^*M337V/+*^ mutation through DRD2‐independent mechanisms. (A) UMAP representation of temporal transcriptome changes in *TARDBP* ^*+/+*^, *TARDBP* ^*M337V/+*^, and *TARDBP* ^*M337V/+*^; *DRD2* ^*−/−*^‐MNs with/without ROPI treatment. (B) Comparison of DEGs in *TARDBP* ^*M337V/+*^ and *TARDBP* ^*M337V/+*^; *DRD2* ^*−/−*^‐MNs at 84 h. (C) KEGG pathway analysis of DEGs specific to DRD2‐dependent pathways in *TARDBP* ^*M337V/+*^. (D) Volcano plot depicting the pairwise analysis between *TARDBP* ^*M337V/+*^; *DRD2* ^*−/−*^ and *TARDBP* ^*+/+*^‐MNs at 84 h. (E) Volcano plot illustrating the pairwise analysis between *TARDBP* ^*M337V/+*^; *DRD2* ^*−/−*^‐MNs with and without ROPI treatment at 84 h. (F) Scatterplot comparing the results of D and E after conversion to directionally adjusted *p* values. (G) GO/KEGG pathway analysis for protective gene sets upregulated by ROPI (top) and downregulated by ROPI (bottom).

We demonstrated that ROPI rescues the ALS cell death, ROS, and neuronal hyperexcitation phenotypes via DRD2‐independent mechanisms. To investigate the DRD2‐independent molecular mechanisms underlying ROPI function, we conducted a time series transcriptomic analysis. We systematically examined the temporal dynamics of gene expression at 6, 36, and 84 h after ROPI treatment in *TARDBP* ^*+/+*^, *TARDBP* ^*M337V/+*^, and *TARDBP* ^*M337V/+*^; *DRD2* ^*−/−*^‐MNs. These time points were chosen to represent distinct phases of the cellular response: an early phase (6 h), a presymptomatic phase before the manifestation of ROS production and cell death (36 h), and a post‐symptomatic phase after the appearance of these phenotypes (84 h). Gene expression in *TARDBP* ^*M337V/+*^‐MNs diverged from that in *TARDBP* ^*+/+*^‐MNs as early as 6 h (Figure 4A). In contrast, gene expression changes in *TARDBP* ^*M337V/+*^; *DRD2* ^*−/−*^‐MNs were minimal compared with those in *TARDBP* ^*M337V/+*^‐MNs. To investigate whether the gene expression changes observed in *TARDBP* ^*M337V/+*^‐MNs are attributable to impaired TARDBP function, we compared our transcriptomic data with publicly available datasets from TARDBP knockdown (KD) studies (Polymenidou et al. 2011). This analysis revealed a substantial overlap between the downregulated genes in TARDBP KD and *TARDBP* ^*M337V/+*^‐MNs (59 out of 93 genes), as well as a smaller overlap among upregulated genes (12 out of 85 genes) (Figure S6). Furthermore, when we examined whether these commonly downregulated genes are direct targets of TARDBP using publicly available CLIP‐seq datasets, we found that 52 out of 59 genes were indeed bound by TARDBP (Polymenidou et al. 2011). These findings suggest that *TARDBP* ^*M337V/+*^‐MNs exhibit a partial loss of TARDBP function. ROPI treatment in *TARDBP* ^*M337V/+*^‐MNs resulted in significant changes in expression at 84 h, which was consistent with the expression pattern of *TARDBP* ^*+/+*^‐MNs. Additionally, ROPI treatment of *TARDBP* ^*M337V/+*^; *DRD2* ^*−/−*^‐MNs for 84 h resulted in a gene expression pattern similar to that of *TARDBP* ^*+/+*^‐MN, which resembled the response of *TARDBP* ^*M337V/+*^‐MNs to ROPI.

To analyze DRD2‐dependent gene expression changes induced by ROPI, we compared the ROPI responsiveness of *TARDBP* ^*M337V/+*^‐MNs and *TARDBP* ^*M337V/+*^; *DRD2* ^*−/−*^‐MNs at 84 h. We also conducted enrichment analysis on the top 0.5% of genes exhibiting differential ROPI responsiveness in *TARDBP* ^*M337V/+*^‐MNs and *TARDBP* ^*M337V/+*^; *DRD2* ^*−/−*^‐MNs (Figure 4B). Differences were observed in pathways such as the ribosome biogenesis (KEGG pathway, *p* = 9.99 × 10^−9^) and the oxidative phosphorylation pathway (KEGG pathway, *p* = 5.93 × 10^−6^; Figure 4C). However, the transcriptomic changes induced by ROPI in *TARDBP* ^*M337V/+*^‐MNs and *TARDBP* ^*M337V/+*^; *DRD2* ^*−/−*^‐MNs were strikingly similar (*p* < 0.001, *R* = 0.898; Figure 4B). These ROPI‐induced changes in gene expression suggested that most genes are regulated by DRD2‐independent mechanisms. We subsequently analyzed which *TARDBP* ^*M337V/+*^ or *TARDBP* ^*M337V/+*^; *DRD2* ^*−/−*^‐MN‐induced gene expression abnormalities were ameliorated by ROPI.

We first identified differentially expressed genes (DEGs) in *TARDBP* ^*M337V/+*^ or *TARDBP* ^*M337V/+*^; *DRD2* ^*−/−*^‐MNs compared to *TARDBP* ^*+/+*^‐MNs (adjusted *p* value < 0.05). In *TARDBP* ^*M337V/+*^ ‐MNs, 2186 genes were upregulated and 1571 were downregulated (Figure S7A). In *TARDBP* ^*M337V/+*^; *DRD2* ^*−/−*^‐MNs, 2441 genes were upregulated and 1471 were downregulated (Figure 4D). We conducted a detailed analysis of the changes in gene expression caused by the DRD2‐independent functions of ROPI. We compared the genes that exhibited alterations in *TARDBP* ^*M337V/+*^ or *TARDBP* ^*M337V/+*^; *DRD2* ^*−/−*^‐MNs with those that were affected by ROPI treatment. A substantial number of genes that showed expression abnormalities in *TARDBP* ^*M337V/+*^ or *TARDBP* ^*M337V/+*^; *DRD2* ^*−/−*^‐MNs were restored to normal levels after ROPI treatment (*TARDBP* ^*M337V/+*^: *p* < 0.001, *R* = −0.627; Figure S7A–C; *TARDBP* ^*M337V/+*^: *DRD2* ^*−/−*^‐MN, *p* < 0.001, *R* = −0.669; Figure 4D–F). To confirm the reliability of our transcriptomic findings, we validated the expression changes of candidate genes identified in *TARDBP* ^*M337V/+*^ and *TARDBP* ^*M337V/+*^; *DRD2* ^*−/−*^‐MNs, as well as those altered by ROPI treatment, using an independent experimental system (Figures S8 and S9). Gene Ontology (GO) enrichment analysis of *TARDBP* ^*M337V/+*^; *DRD2* ^*−/−*^‐MNs highlighted improvements in the expression levels of genes in GO categories related to protein synthesis, including cytoplasmic translation (GO: biological process [BP], *p* = 1.30 × 10^−40^) and ribosomal subunit (GO: cellular component [CC], *p* = 2.53 × 10^−39^), as well as among genes related to energy metabolism, such as oxidoreduction‐driven active transmembrane transporter activity (GO: molecular function [MF], *p* = 4.41 × 10^−4^) and oxidative phosphorylation (KEGG, *p* = 1.82 × 10^−8^) (Figure 4G). The GO categories of the set of genes recovered by ROPI in *TARDBP* ^*M337V/+*^; *DRD2* ^*−/−*^‐MNs showed substantial overlap with those recovered by ROPI in *TARDBP* ^*M337V/+*^‐MNs (Figure 4G, Figure S7D). Additionally, we performed an analysis of candidate genes associated with the reduction of cell death and the suppression of neuronal hyperexcitability by ROPI. The results revealed that the DRD2‐independent action of ROPI improved the expression of genes involved in ferroptosis, including *GPX4*, *ferritin*, *transferrin receptor* (*TFRC*), and *divalent metal transporter 1* (*DMT1*) (Table S6). Furthermore, ROPI corrected the expression of voltage‐gated sodium channels, including *SCN3A*, *SCN5A*, and *SCN8A*, which are associated with neuronal hyperexcitability (Table S7). These data suggest that the resolution of abnormal gene expression by ROPI through a DRD2‐independent mechanism may be involved in phenotypic improvement.

### 3.7 ROPI Rescues Aberrant mRNA Splicing in *TARDBP* ^*M337V/+*^‐MNs and Its Underlying Mechanisms

**FIGURE 5:**
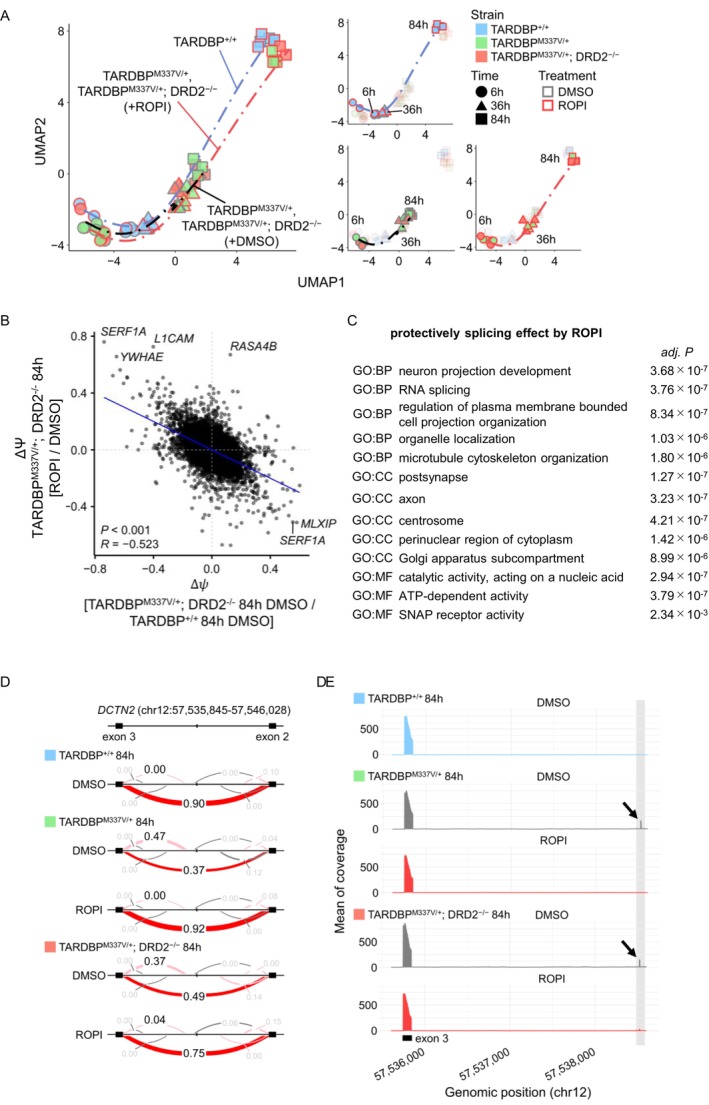
ROPI rescues aberrant RNA splicing caused by the *TARDBP* ^*M337V/+*^ mutation through DRD2‐independent mechanisms. (A) UMAP depicting the whole‐RNA splicing patterns. (B) Scatterplot comparing the results of pairwise analysis between *TARDBP* ^*M337V/+*^; *DRD2* ^*−/−*^ and *TARDBP* ^*+/+*^ at 84 h and the analysis of *TARDBP* ^*M337V/+*^; *DRD2* ^*−/−*^ under ROPI/DMSO treatment at 84 h after conversion to directionally adjusted *p* values. (C) GO/KEGG pathway analysis of the gene sets with splicing protected by ROPI. (D, E) Schematic diagram depicting splicing changes in DCTN2.

We systematically examined the temporal dynamics of RNA splicing after ROPI treatment in *TARDBP* ^*+/+*^, *TARDBP* ^*M337V/+*^, and *TARDBP* ^*M337V/+*^; *DRD2* ^*−/−*^‐MNs. Compared with that of *TARDBP* ^+/+^‐MNs, the splicing pattern of *TARDBP* ^*M337V/+*^‐MNs changed at 36 h and exhibited significant alterations at 84 h (Figure 5A). In contrast, the changes in RNA splicing in *TARDBP* ^*M337V/+*^; *DRD2* ^*−/−*^‐MNs were minimal compared with those in *TARDBP* ^*M337V/+*^‐MNs. However, treatment with *TARDBP* ^*M337V/+*^‐MNs resulted in significant splicing changes at 84 h, which aligned with the splicing pattern of *TARDBP* ^*+/+*^‐MNs. Additionally, ROPI treatment of *TARDBP* ^*M337V/+*^; *DRD2* ^*−/−*^‐MNs for 84 h resulted in a splicing pattern similar to that of *TARDBP* ^*+/+*^‐MNs, resembling the response of *TARDBP* ^*M337V/+*^‐MNs to ROPI. We compared the genes that exhibited alterations in RNA splicing in *TARDBP* ^*M337V/+*^‐MNs or *TARDBP* ^*M337V/+*^; *DRD2* ^*−/−*^‐MNs with those affected by ROPI treatment. A substantial number of genes showing splicing abnormalities due to *TARDBP* ^*M337V/+*^‐MNs or *TARDBP* ^*M337V/+*^; *DRD2* ^*−/−*^‐MNs were restored to normal levels after ROPI treatment (*TARDBP* ^*M337V/+*^; *DRD2* ^*−/−*^‐MNs: *p* < 0.001, *R* = −0.523; Figure 5B, *TARDBP* ^*M337V/+*^‐MNs: *p* < 0.001, *R* = −0.516; Figure S10A). To further investigate the effects of ROPI, we focused on the overlap between splicing abnormalities observed in both *TARDBP* ^M337V/+^ and *TARDBP* ^M337V/+^; *DRD2* ^−/−^‐MNs and examined how ROPI treatment influenced these gene groups. We also identified a group of genes whose splicing is protected by ROPI: 456 genes in *TARDBP* ^M337V/+^ MNs were restored by ROPI, and 486 genes in *TARDBP* ^M337V/+^; *DRD2* ^−/−^‐MNs were similarly restored by ROPI. To better understand the biological significance of the genes whose splicing abnormalities were restored by ROPI, we conducted a GO enrichment analysis. GO enrichment analysis of this group highlighted amelioration of splicing abnormalities in genes with annotations associated with RNA splicing (GO:BP, *p* = 3.76 × 10^−7^), as well as catalytic activity, acting on a nucleic acid (GO:MF, *p* = 2.94 × 10^−7^), and in genes with annotations related to neurite function, such as post‐synapse (GO:CC, *p* = 1.27 × 10^−7^) and axon (GO:CC, *p* = 3.23 × 10^−7^), which includes genes annotated with mitochondrial function terms, such as ATP‐dependent activity (GO:MF, *p* = 3.91 × 10^−5^, Figure 5C, Figure S10B). The GO categories enriched among genes recovered by ROPI in *TARDBP* ^*M337V/+*^; *DRD2* ^*−/−*^‐MNs showed substantial overlap with those recovered by ROPI in *TARDBP* ^*M337V/+*^‐MNs (Figure 5C, Figure S10B).

We identified *DCTN2* as a gene with splicing changes suggesting its involvement in ALS (Figure 5D,E). DCTN2 binds to dynein and is associated with axonal transport, a process that is a known aspect of ALS pathology (De Vos and Hafezparast 2017; LaMonte et al. 2002). Splicing linking Exons 2 and 3 in *DCTN2* was observed at a rate of 90% in *TARDBP* ^*+/+*^‐MNs but decreased to 37% in *TARDBP* ^*M337V/+*^‐MNs and 49% in *TARDBP* ^*M337V/+*^; *DRD2* ^*−/−*^‐MNs. Instead, a new splicing event occurred, involving the junction between Exons 2 and 3 (0% in *TARDBP* ^*+/+*^‐MN, 47% in *TARDBP* ^*M337V/+*^‐MNs, and 37% in *TARDBP* ^*M337V/+*^; *DRD2* ^*−/−*^‐MN; Figure 5D,E). This alternative splicing pattern was rarely observed following treatment with ROPI, in line with the observations in *TARDBP* ^*+/+*^‐MNs (0% in *TARDBP* ^*M337V/+*^‐MNs and 4% in *TARDBP* ^*M337V/+*^; *DRD2* ^*−/−*^‐MN; Figure 5D,E). To predict the functional impact of this splicing event, structural modeling was performed. The splicing defect provided a novel alpha helix structure into the N‐terminal region of DCTN2, which may also lead to structural distortions extending toward the C‐terminal region (Figure S12). These data suggest that the resolution of aberrant mRNA splicing by the DRD2‐independent mechanism of ROPI may contribute to phenotypic improvement in ALS.

## 4 Discussion

We previously reported that ROPI suppresses ALS phenotypes such as apoptosis, neurite retraction, and protein aggregation. We previously suggested the presence of a DRD2‐independent mechanism on the basis of analyses using a DRD2 antagonist; however, we were unable to reach a definitive conclusion (Fujimori et al. 2018; Morimoto et al. 2023). In this study, we investigated whether the therapeutic effects of ROPI in ALS are dependent on DRD2. To clarify this dependency, we used CRISPR/Cas9 genome‐editing technology to establish isogenic iPSCs with *DRD2* gene deficiency by combining isogenic *TARDBP*‐mutant iPSCs. In *DRD2* knockout *TARDBP*‐mutant MNs, ROPI suppressed cell death, oxidative stress, and neuronal hyperexcitation, similar to its effects on DRD2 wild‐type ALS‐MNs. Transcriptomic analysis revealed that ROPI rescued aberrant mRNA splicing and abnormal gene expression.

### 4.1 The Protective Effect of ROPI Against Cell Death May Be Mediated Through the Inhibition of ROS

We demonstrated that ROPI suppresses neuronal cell death through a DRD2‐independent mechanism. Cell death in ALS is partly due to increased ROS levels (Cunha‐Oliveira et al. 2020; Motataianu et al. 2022). Building on our previous research and the current findings, we propose three potential mechanisms by which the DRD2‐independent action of ROPI may reduce ROS levels and mitigate neuronal cell death. Three potential mechanisms by which the DRD2‐independent action of ROPI may reduce ROS levels are proposed: (1) a mechanism mediated by the Fenton reaction, (2) a mitochondrial‐dependent mechanism, and (3) a mechanism involving the structural properties of ROPI. Further details of these mechanisms will be presented later.

The first potential mechanism involves the Fenton reaction. The Fenton reaction contributes to an increase in ROS levels by generating highly reactive hydroxyl radicals (˙OH) through the reaction of hydrogen peroxide (H_2_O_2_) with ferrous iron (Fe^2+^) (Lee and Hyun 2023). This process amplifies oxidative stress within the cell, leading to potential damage to lipids, proteins, and DNA. The Fenton reaction culminates in cell death via ferroptosis, a newly defined iron‐dependent cell death. In this study, transcriptome analysis revealed that the expression of several ferroptosis‐related genes, including GPX4, ferritin, transferrin receptor (TFRC), and divalent metal transporter 1 (DMT1), was dysregulated in *TARDBP* ^M337V/+^; *DRD2* ^−/−^‐MNs. Reduced ferritin levels and increased expression of TFRC and DMT1 promote the Fenton reaction by increasing ferrous iron levels, thereby predisposing cells to ferroptosis (Battaglia et al. 2020; Hintze and Theil 2006; Anderson and Frazer 2017). Notably, treatment with ROPI restored the expression levels of these genes. Moreover, we previously reported that the population responsive to ROPI exhibited increased levels of lipid peroxidation, a marker of ferroptosis, which were reduced following ROPI treatment (Fujimori et al. 2018). While these findings indicate a potential association between ROPI treatment and transcriptional regulation of ferroptosis‐related genes, a direct causal link between ROPI and the modulation of these pathways remains to be elucidated. These results suggest that the DRD2‐independent manner of ROPI may inhibit cell death by reducing ROS levels through the modulation of ferroptosis‐related gene expression.

Another potential mechanism is the mitochondrial‐dependent pathway. Elevated ROS levels have been observed in ALS‐MNs, with reported causative factors including mitochondrial abnormalities, elevated NADPH oxidase activity and expression, and irregularities in the NRF2 pathway (Cunha‐Oliveira et al. 2020; Motataianu et al. 2022). In this study, elevated ROS levels in *TARDBP* ^*M337V/+*^‐MNs were alleviated by ROPI in a D2R‐independent manner. In addition, transcriptomic analysis revealed alterations in mitochondrial oxidative phosphorylation pathways. This pathway assumes a critical role in metabolically active cells, such as MNs, which underscores the importance of energy metabolism and mitochondrial function. mRNA destabilization in ALS‐MNs, which is related to oxidative phosphorylation, leads to decreased protein expression, as observed in autopsy studies of patients with ALS (Tank et al. 2018). Tank et al. demonstrated that mitochondrial and ribosomal transcripts are among the most destabilized mRNAs in ALS motor neurons. These transcripts contribute to cellular dysfunction through impaired bioenergetics and proteostasis. Moreover, a reduction in the expression of NADH dehydrogenase, a factor related to oxidative phosphorylation, has been reported to result in increased mitochondria‐derived ROS in patients with ALS (Tsai et al. 2020). These previous studies, along with our research, suggest that ROPI may increase mRNA expression during oxidative phosphorylation through DRD2‐independent actions and consequently suppress ROS production.

Finally, the structural properties of ROPI may contribute to its ability to reduce ROS. The DRD2‐independent anti‐ALS activity of ROPI could also be attributed to its distinctive structural and chemical characteristics (Okano et al. 2020). Structurally, ROPI has an oxindole (indoline‐2‐one) skeleton and an *N*,*N*‐di‐*n*‐propylethylamine moiety. The oxindole backbone of ROPI is the chemical equivalent of phenol, which is a general antioxidant. While oxindole itself does not exhibit greater antioxidant activity than other antioxidants, such as uric acid (Ferrari‐Toninelli et al. 2010), reports indicate that ROPI effectively scavenges ROS (Yasuda et al. 2013). Notably, other dopamine receptor agonists, such as rotigotine, bromocriptine, and pergolide, lack an oxindole skeleton. This structural distinction may contribute to the inhibition of ROS production by ROPI, explaining its therapeutic effects on ALS. Additionally, under physiological pH conditions, the tertiary amine segment of ROPI exists as a lipophilic cation (Yasuda et al. 2013; Okano et al. 2020), which facilitates its easy passage through the cell membrane. Consequently, ROPI selectively targets and localizes to the strongly negatively charged inner mitochondrial membrane (Murphy 2008). Therefore, ROPI readily localizes to the mitochondria, possibly by scavenging ROS to protect the mitochondria and prevent cell death. The properties of dexpramipexole are similar to those of ROPI. In addition to its DRD2 agonist activity, dexpramipexole has structural antioxidant properties and the ability to accumulate in mitochondria (Wang et al. 2008). While phase 3 clinical trials of dexpramipexole did not demonstrate its efficacy in patients with ALS, effectiveness was observed in subgroup analyses and in phase 2 trials (Cudkowicz et al. 2013; Bozik et al. 2014). In contrast, the general DRD2 agonist bromocriptine was not found to be effective in phase 2 trials (Nagata et al. 2016). The limited efficacy of riluzole and edaravone, along with the failure of many clinical trials, suggests that combination therapies or multitarget compounds may be more effective than monotherapies. At least in vitro, the anti‐ALS effect of ROPI appears to be superior to those of the DRD2 agonists dexpramipexole and pramipexole (Fujimori et al. 2018). Collectively, these reports indicate that one of the DRD2‐independent mechanisms of ROPI is its structural antioxidant effects on mitochondria. The multifunctional properties of ROPI suggest a reason for its greater clinical utility compared to other compounds.

### 4.2 Hyperexcitation Is Suppressed by ROPI Through Both DRD2‐Dependent and DRD2‐Independent Mechanisms

Longitudinal MEA recordings demonstrated that *TARDBP* ^M337V/+^ and *TARDBP* ^M337V/+^; *DRD2* ^−/−^‐MNs exhibited increasing spontaneous activity starting from Day 9, whereas control MNs remained largely inactive. This observation is consistent with previous studies showing that iPSC‐derived wild‐type motor neurons generally display low or undetectable levels of spontaneous firing under standard culture conditions in the absence of excitatory factors (Morimoto et al. 2023; Kondo et al. 2023). These results further support that spontaneous hyperexcitability is a disease‐intrinsic feature of ALS motor neurons.

In previous studies, DRD2 agonists inhibited spontaneous hyperexcitability because DRD2 signaling induces a reduction in cAMP levels and the activation of G protein‐gated inwardly rectifying K(+) (GIRK) channels (Huang et al. 2021; Morimoto et al. 2023). In the present study, ROPI inhibited the hyperexcitability of DRD2‐deficient MNs. Abnormalities in ion channels, such as sodium channel conductance, contribute to the development of membrane hyperexcitability in ALS. This leads to the generation of symptoms such as muscle cramps and fasciculations and promotes a neurodegenerative cascade via Ca^2+^‐mediated processes (Park et al. 2017; Saba et al. 2019). Transcriptomic analysis demonstrated that the expression of specific voltage‐gated sodium channels, including SCN3A, SCN5A, and SCN8A, was increased in *TARDBP* ^*M337V/+*^; *DRD2* ^*−/−*^‐MNs. Furthermore, the addition of ROPI resulted in decreased expression of these genes. These results suggest that sodium channels contribute to the inhibition of excitotoxicity, independent of DRD2. In contrast, the inhibitory effect of ROPI on hyperexcitability in *DRD2*‐deficient MNs was reduced compared with that of ROPI in MNs with DRD2. These results suggest that ROPI contributes to the inhibition of neural hyperexcitability through both DRD2‐mediated cAMP inhibition and non‐DRD2‐mediated pathways.

### 4.3 The Improvement in ALS Phenotypes by ROPI May Be due to Its Effects on Splicing Correction via DRD2 Independent Mechanism

Splicing abnormalities have been reported in association with *TARDBP* knockdown and mutation in patients with ALS (Cao et al. 2023; Klim et al. 2019; Ma et al. 2022; Melamed et al. 2019). Loss of TDP‐43 protein from the nucleus in iPSC‐derived MNs increased cryptic exon splicing of *UNC13A* and *STMN2* mRNAs (Ma et al. 2022; Klim et al. 2019). The results of this study revealed minimal cryptic exon splicing of *STMN2* in *TARDBP* ^*M337V/+*^‐MNs (Figure S11). However, we confirmed that splicing abnormalities in many mRNAs were also observed in *TARDBP* ^*M337V/+*^‐MNs. Interestingly, these abnormalities were reversed through a DRD2‐independent mechanism upon treatment with ROPI.

One ALS‐related gene whose splicing abnormalities were ameliorated by ROPI was DCTN2, a critical subunit of the dynactin complex. The dynactin complex works in conjunction with dynein to facilitate intracellular transport along microtubules, including axonal transport in neurons (Reck‐Peterson et al. 2018). Disruptions in axonal transport, a critical process for maintaining neuronal homeostasis, have been implicated as a key factor in the pathogenesis of neurodegenerative diseases, including Alzheimer's disease, Parkinson's disease, and ALS (LaMonte et al. 2002; Cason and Holzbaur 2022). This study identified aberrant splicing in the N‐terminal region of DCTN2 in *TARDBP* ^*M337V/+*^‐MN. The splicing defect provided a novel alpha helix structure into the N‐terminal region of DCTN2, which may also lead to structural distortions extending toward the C‐terminal region. This N‐terminal region is critical for directly binding the cargo‐binding Arp1 filament, a key component of the dynactin complex, which is essential for maintaining dynactin's structural integrity (Cheong et al. 2014). Taken together, these findings suggest that ROPI's ameliorating effect on DCTN2 splicing abnormalities may improve ALS phenotypes by enhancing axonal transport functionality through the DCTN2‐Arp1 interaction. Future studies will focus on axonal transport assays and interaction analyses with Arp1 to validate the hypothesis. Specifically, we plan to investigate the role of the abnormal DCTN2 protein in axonal transport and its interactions with Arp1 within the dynactin complex. Additionally, we aim to explore how ROPI ameliorates these splicing abnormalities and restores axonal transport functionality, potentially improving interactions within the dynactin complex and mitigating defects linked to ALS phenotypes.

Collectively, these findings suggest that ROPI might alleviate the ALS phenotype by addressing splicing abnormalities. While its involvement in splicing regulation has been demonstrated, the precise mechanisms underlying ROPI's role remain unclear. Moreover, the functional consequences of protein variants generated through alternative splicing in response to ROPI treatment require further elucidation.

## 5 Limitations, Conclusions, and Perspectives

We are mindful of the limitations of our study. This study investigated the therapeutic effects of ROPI in DRD2‐independent pathways using *TARDBP* ^M337V^ mutant MNs. However, the majority of patients with ALS have sporadic ALS, and this patient population is highly heterogeneous (Fujimori et al. 2018; Okano and Morimoto 2022). Therefore, to validate the efficacy of the DRD2‐independent effects of ROPI in ALS, it is necessary to conduct analyses using motor neurons derived from familial ALS patients with different mutations and a large cohort of sporadic ALS patients. In particular, we recently found that suppression of the SREBP2‐dependent cholesterol biosynthetic pathway may be involved in the therapeutic effect of ROPI in SALS patients (Morimoto et al. 2023; Okano et al. 2023; Kato et al. 2024). However, we do not know whether such an effect of ROPI on cholesterol metabolism is DRD2‐dependent or not. Further research is also necessary to demonstrate that the ability of ROPI to resolve changes in the expression of oxidative phosphorylation‐related genes and numerous splicing abnormalities ameliorates ALS pathogenesis. Additionally, although highly challenging, it will be important to validate the direct targets of ROPI and elucidate the detailed molecular mechanisms that control the resolution of these expression and splicing abnormalities to advance our understanding of ALS therapies.

Overall, this study provides insights into the DRD2‐independent effects of ROPI on cell death, ROS levels, and hyperexcitability in the context of isogenic iPSC generation, MN induction, and *DRD2* knockout in *TARDBP* mutants. Furthermore, we suggest that ROPI ameliorates the ALS phenotype by resolving splicing and gene expression abnormalities via a DRD2‐independent mechanism. Ultimately, our study offers promising avenues for the development of effective therapies that could significantly affect the lives of patients with ALS and brings the field closer to new innovative and targeted interventions.

## Author Contributions

**Hirotsugu Asano:** conceptualization, investigation, data curation, formal analysis, validation, visualization, writing – original draft, writing – review and editing. **Tetsuya Kawaguchi:** investigation, data curation, formal analysis, resources, validation, visualization, writing – original draft, writing – review and editing. **Chris Kato:** formal analysis, visualization, writing – original draft. **Satoru Morimoto:** formal analysis, validation, writing – review and editing. **Masato Yano:** data curation, formal analysis, visualization. **Maki Minaguchi:** investigation, data curation. **Daisuke Yasuda:** resources. **Komei Fukushima:** project administration. **Hideyuki Okano:** conceptualization, project administration, supervision, validation, writing – review and editing.

## Conflicts of Interest

H.A., T.K. and M.M. are employees of K Pharma Inc. H.O. is the founder and Chief Scientific Officer of K Pharma Inc. K.F. was the founder and Chief Executive Officer of K Pharma Inc. The remaining authors declare no conflicts of interest.

## Supporting information


**Appendix S1:** jnc70183‐sup‐0001‐AppendixS1.zip.

## Data Availability

The RNA‐seq data have been deposited at the Gene Expression Omnibus (GEO) and are publicly available as of the date of publication. GEO accession number is as follows: GSE283507.
