# Supplementary material for: Ropinirole Functions Through a Dopamine Receptor D2‐Independent Mechanism to Ameliorate Amyotrophic Lateral Sclerosis Phenotypes in TARDBP ‐Mutant iPSC‐Derived Motor Neurons
Source: J Neurochem. 2025 Aug 18;169(8):e70183. doi: 10.1111/jnc.70183 (PMC12361731; doi:10.1111/jnc.70183)
Supplement: Supplementary file 1 — Appendix S1: jnc70183‐sup‐0001‐AppendixS1.zip. [file JNC-169-0-s001.zip › Supplementary Data.zip!/Supplementary Data/Asano_et_al_JNeurochem_Supplement_Revised.pdf]

Supplementary Information

Ropinirole functions through a dopamine receptor D2-independent mechanism to ameliorate amyotrophic lateral sclerosis phenotypes in *TARDBP*-mutant iPSC-derived motor neurons

Hirotsugu Asano,[1] Tetsuya Kawaguchi,[1] Chris Kato,[2] Satoru Morimoto,[2] Daisuke Yasuda,[3] Komei Fukushima,[1] Hideyuki Okano[1,2] *

[1]R&D Department, K Pharma, Inc., Kanagawa, Japan

[2]Keio University Regenerative Medicine Research Center, Kanagawa, Japan

[3]Department of Pharmaceutical Sciences, Osaka Medical and Pharmaceutical University, Osaka, Japan

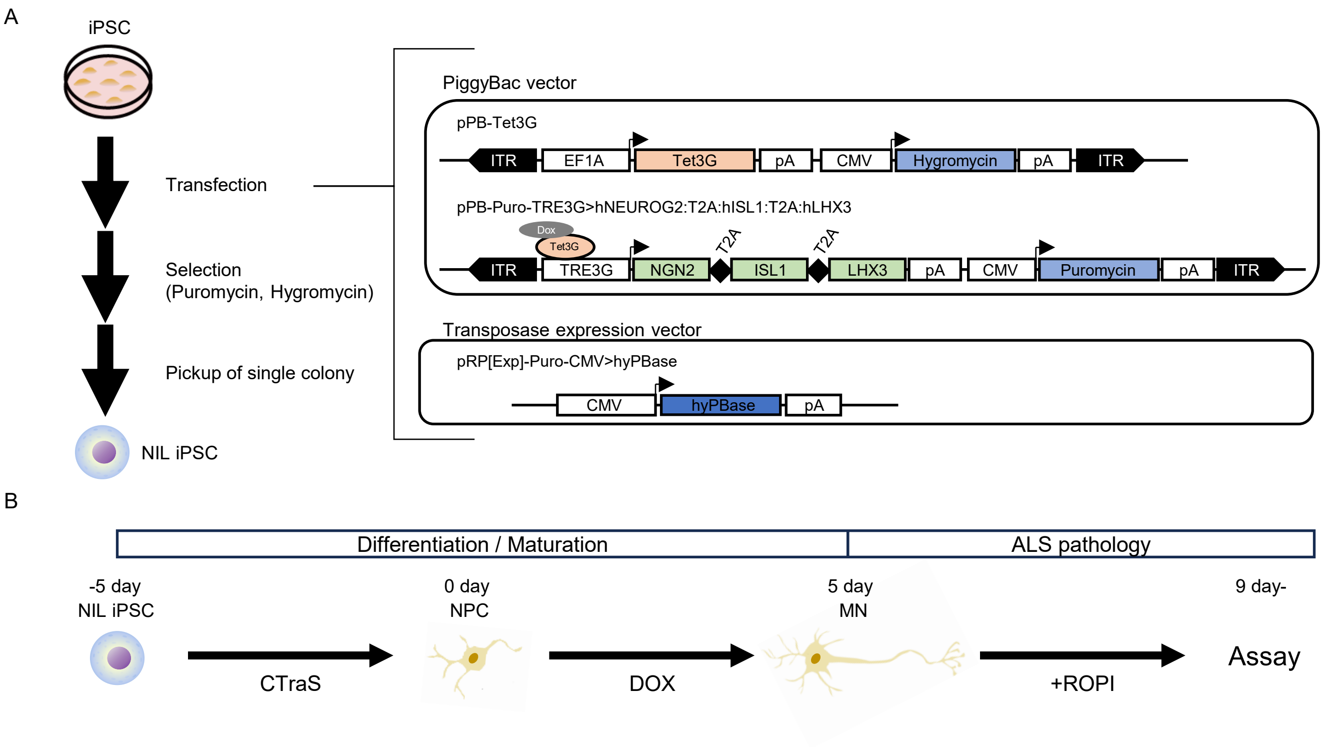

SUPPLEMENTARY FIGURE 1

Characterization of TARDBP$^{M337V/+}$; DRD2$^{-/-}$-MN. A. Schematic diagram of iPSC generation. B. Schematic diagram of the culture conditions for MNs.

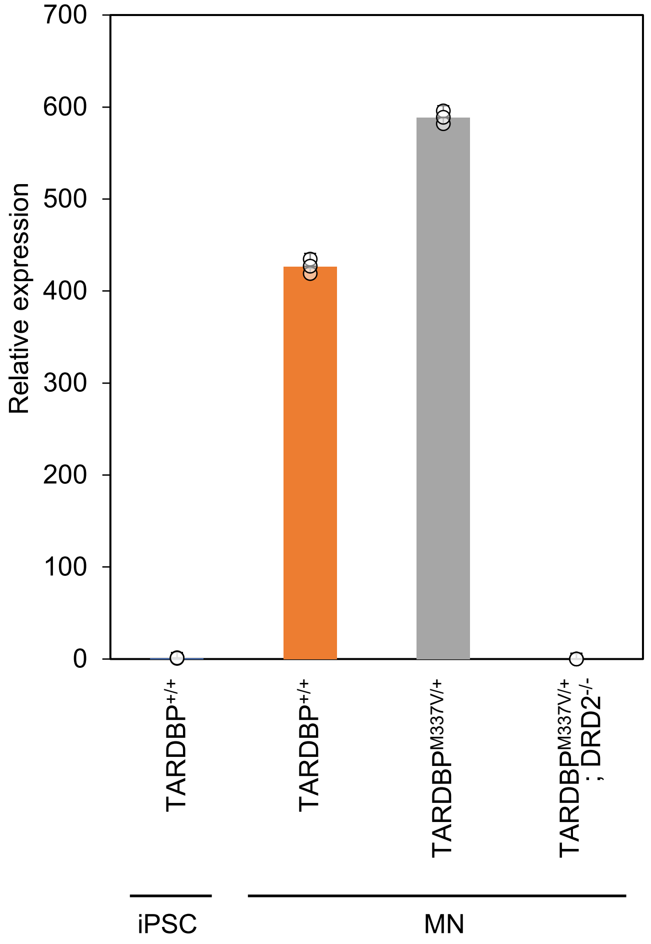

SUPPLEMENTARY FIGURE 2

Individual data plots for DRD2 mRNA expression in $TARDBP^{M337V/+}$-MN and $TARDBP^{M337V/+}$; $DRD2^{-/-}$-MN. Each data point represents an individual biological replicate (n = 3 per group). Mean ± SD is indicated by horizontal lines.

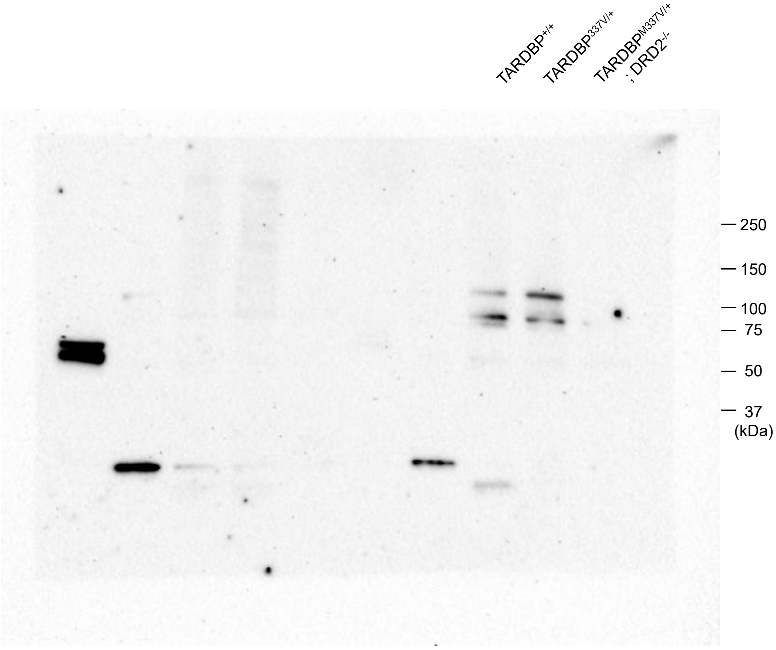

SUPPLEMENTARY FIGURE 3

Original data from immunoblotting analysis of DRD2 protein expression in $TARDBP^{M337V/+}$-MN and $TARDBP^{M337V/+}$; $DRD2^{-/-}$-MN. Lanes 9–11 were probed with anti-DRD2 antibody.

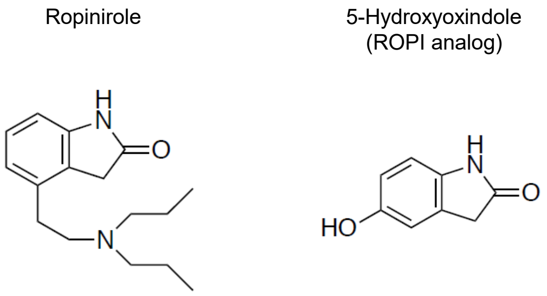

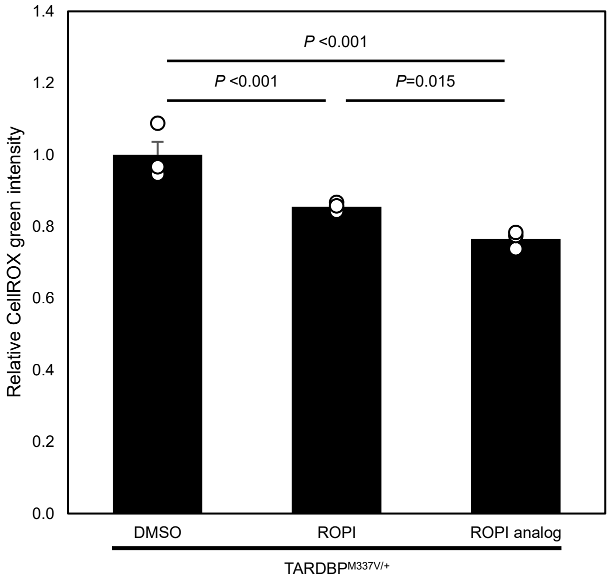

SUPPLEMENTARY FIGURE 4

Left: Structure of ROPI and the 5-hydroxyoxindole of the ROPI analog with a deleted DRD2-binding region. Right: Inhibition of ROS by a ROPI analog in TARDBP$^{M337V/+}$-MN.

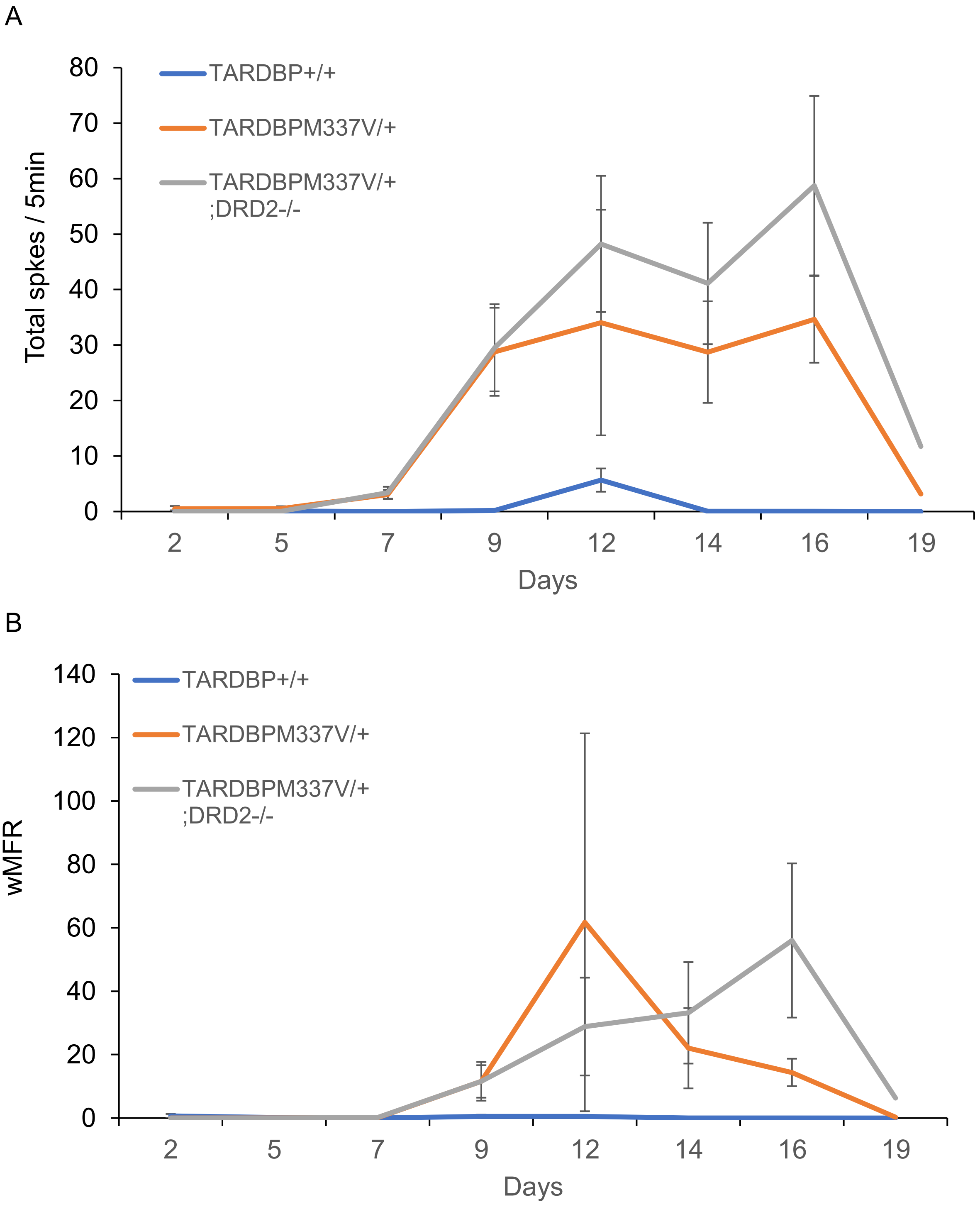

SUPPLEMENTARY FIGURE 5

MNs with TARDBP$^{M337V/+}$ mutation display spontaneous neuronal hyperexcitability. A,B, The spontaneous firing on multielectrode array (MEA) in the human iPSC-derived MNs with TARDBP$^{+/+}$ (blue line), TARDBP$^{M337V/+}$(orange line) and TARDBP$^{M337V/+}$; DRD2$^{-/-}$ (grey line) , shown as the total number of spikes over a 5-minute recording period (A) and the weighted mean firing rate (wMFR) (B). Data are presented as the mean ± SEM (n = 4 independent experiments; 6–9 wells per condition).

A

Expressed genes (Asano et al.)
(8229)
↓
Changed genes (Asano et al.)
(2483)
↓
Down-regulated genes (Asano et al.)
(1569)

Down-regulated genes (Polymenidou et al.)
(239)
↓
Down-regulated genes (Polymenidou et al.)
<Lift_Human gene>
(178)
↓
Down-regulated genes (Polymenidou et al.)
<Lift_Human gene&include 8229 gene>
(93)
↓
Down-regulated genes (Polymenidou et al.)
<Lift_Human gene&include 8229 gene>
</Asano down-regulated 1569>
(59)
↓
Down-regulated genes (Polymenidou et al.)
<Lift_Human gene&include 8229 gene>
</Asano down-regulated 1569>
<Containing CLIP cluster, at least 1>
(52)

B

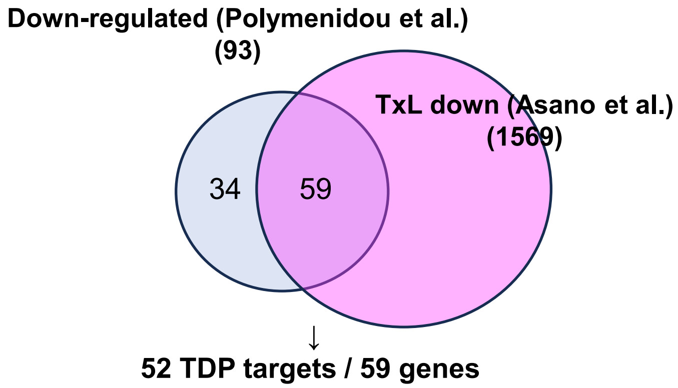

SUPPLEMENTARY FIGURE 6

Comparison of gene expression between TARDBP knockdown cells and TARDBP-M337V mutants. A. Stepwise Gene Filtering for Venn Analysis in Transcriptome Data. B. Venn diagram analysis was conducted to identify genes commonly altered by both TARDBP knockdown (KD) and the M337V TDP-43 mutant. Differentially expressed genes were defined as those showing at least a 2-fold increase or a 50% decrease (fold change ≥ 2 or ≤ 0.5). The left panel shows commonly downregulated genes, while the right panel shows commonly upregulated genes. Among the commonly dysregulated genes, the number of direct TDP-43 targets was determined using publicly available CLIP-Seq datasets.

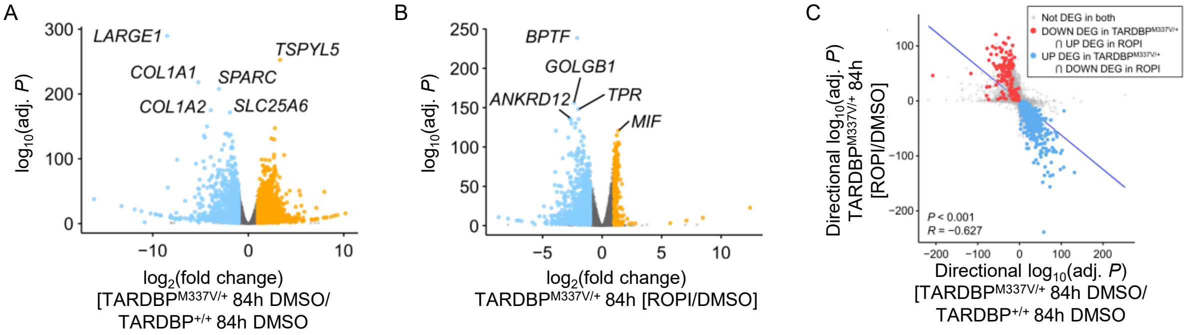

D

Protectively upregulated by ROPI		adj. *P*
GO:MF	rRNA binding	3.09×10^{-06}
GO:MF	proton transmembrane transporter activity	1.24×10^{-05}
GO:MF	oxidoreduction-driven active transmembrane transporter activity	2.51×10^{-05}
GO:BP	cytoplasmic translation	8.10×10^{-30}
GO:BP	aerobic electron transport chain	7.90×10^{-08}
GO:BP	mitochondrial ATP synthesis coupled electron transport	2.51×10^{-07}
GO:CC	cytosolic ribosome	4.10×10^{-38}
GO:CC	ribosomal subunit	1.27×10^{-28}
GO:CC	cytosolic large ribosomal subunit	2.97×10^{-19}
KEGG	Ribosome	1.40×10^{-26}
KEGG	Coronavirus disease - COVID-19	7.47×10^{-21}
KEGG	Parkinson disease	6.12×10^{-10}

Protectively downregulated by ROPI		adj. *P*
GO:MF	histone modifying activity	1.93×10^{-05}
GO:MF	catalytic activity, acting on DNA	2.76×10^{-05}
GO:MF	histone H3 methyltransferase activity	5.01×10^{-05}
GO:BP	centrosome cycle	7.80×10^{-04}
GO:BP	microtubule organizing center organization	8.18×10^{-04}
GO:BP	heterochromatin organization	5.58×10^{-03}
GO:CC	centriole	3.72×10^{-09}
GO:CC	centriolar satellite	1.96×10^{-04}
GO:CC	histone deacetylase complex	3.19×10^{-04}
KEGG	Lysine degradation	6.14×10^{-04}

SUPPLEMENTARY FIGURE 7

ROPI rescues abnormal gene expression caused by the $TARDBP^{M337V/+}$ mutation.

A. Volcano plot depicting the results of the pairwise analysis between $TARDBP^{M337V/+}$ and $TARDBP^{+/+}$ at 84 h. B. Volcano plot illustrating the results of pairwise analysis between $TARDBP^{M337V/+}$, with and without ROPI treatment at 84 h. C. Scatterplot comparing the results of Supplementary Figure 7A and 7B after conversion to directional adjusted P-values. D. GO/KEGG pathway analyses for gene sets protective downregulated by ROPI and protective upregulated by ROPI in $TARDBP^{M337V/+}$-MNs.

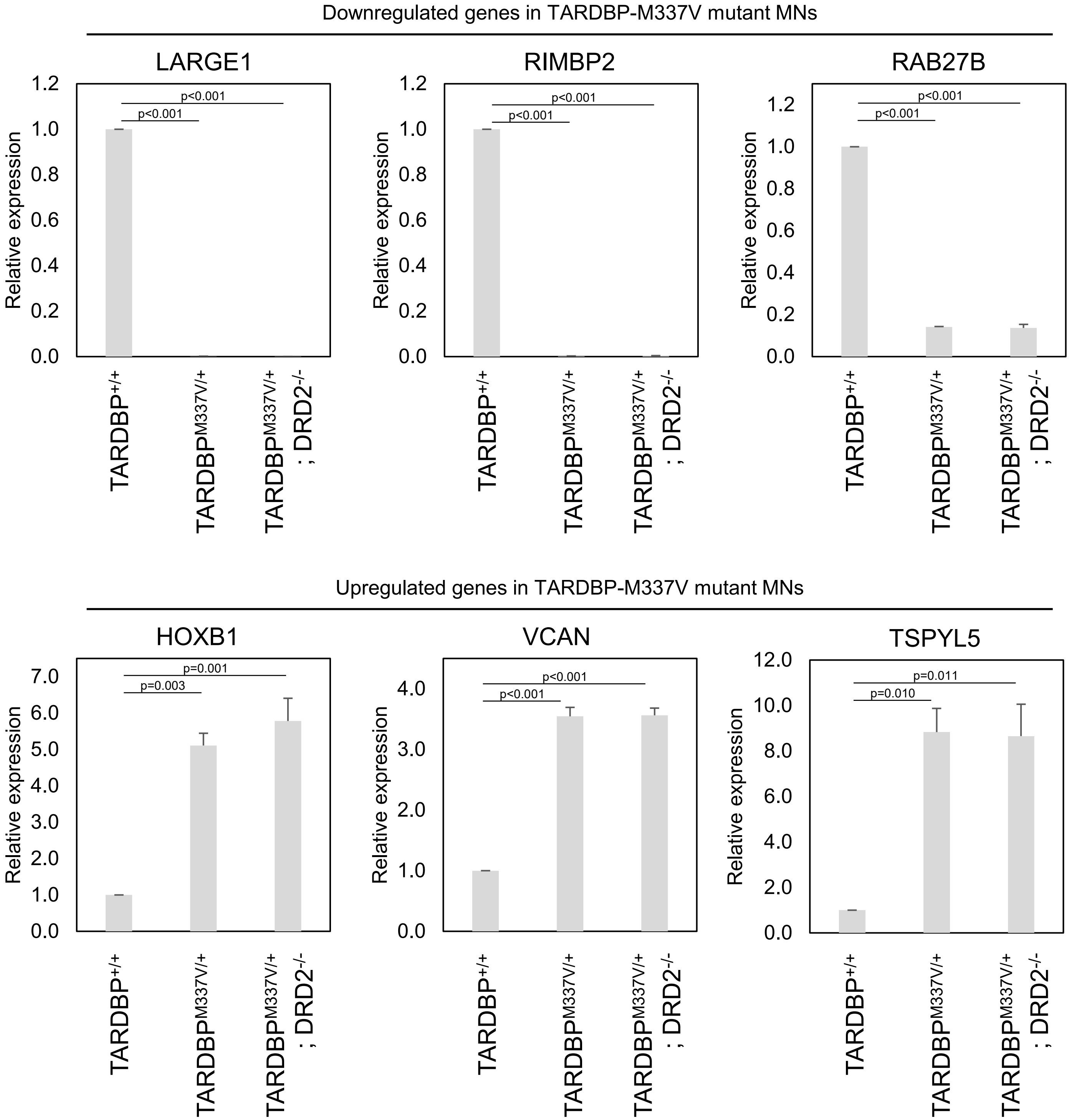

SUPPLEMENTARY FIGURE 8

Validation of changes in gene expression identified by transcriptome analyses.

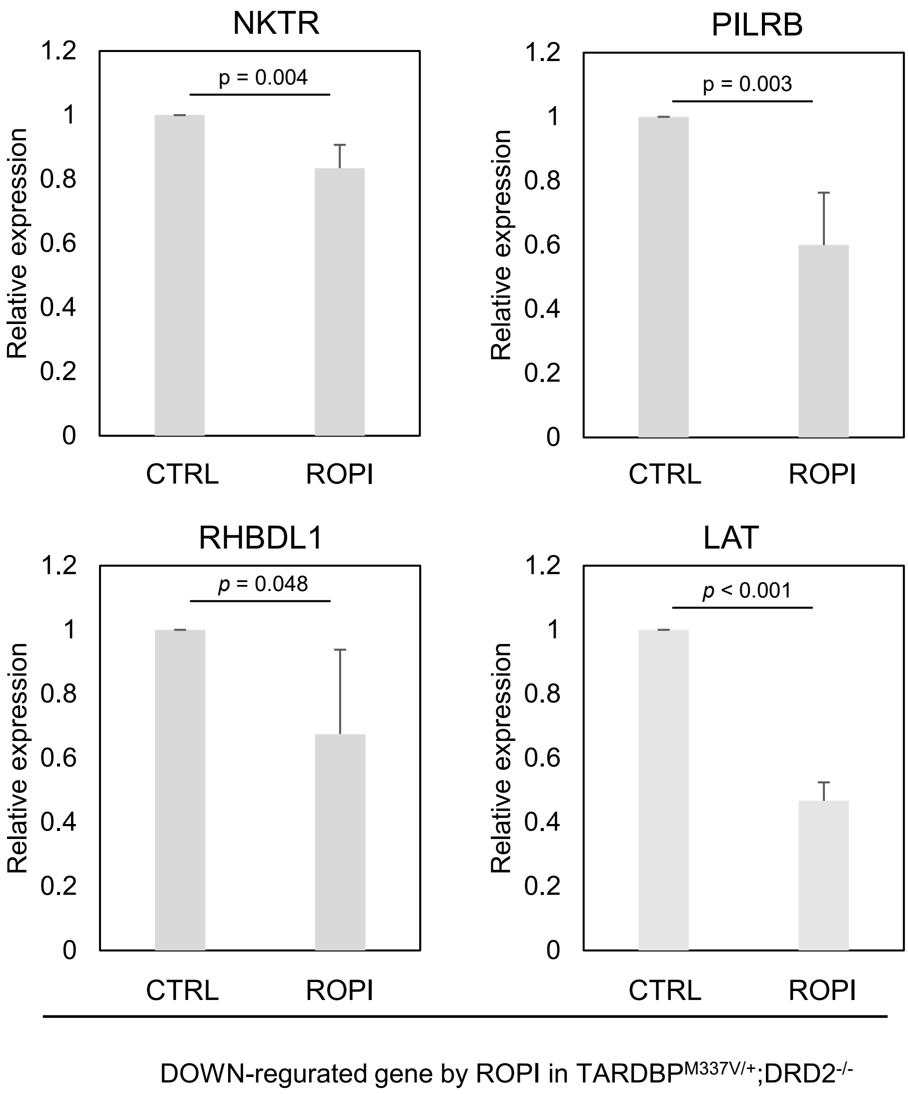

SUPPLEMENTARY FIGURE 9

Validation of Ropinirole-regulated gene expression identified by transcriptome analyses.

A

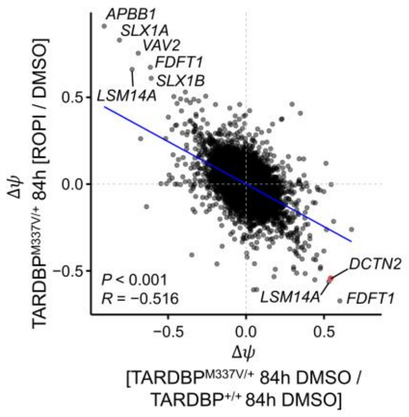

B

protectively splicing effect by ROPI

		adj. P
GO:BP	RNA splicing	7.59×10^{-6}
GO:BP	microtubule cytoskeleton organization	1.97×10^{-5}
GO:BP	regulation of plasma membrane bounded cell projection organization	2.19×10^{-5}
GO:BP	regulation of cell growth	2.21×10^{-5}
GO:BP	cytoskeleton-dependent intracellular transport	2.83×10^{-5}
GO:CC	presynaptic active zone	1.89×10^{-6}
GO:CC	nuclear speck	3.36×10^{-5}
GO:CC	postsynaptic specialization	5.25×10^{-5}
GO:CC	axon	7.29×10^{-5}
GO:CC	granular component	9.62×10^{-5}
GO:MF	catalytic activity, acting on a nucleic acid	3.60×10^{-6}
GO:MF	ATP-dependent activity	3.90×10^{-5}
GO:MF	histone H4 methyltransferase activity	1.40×10^{-3}
GO:MF	SNAP receptor activity	1.85×10^{-3}
GO:MF	kinesin binding	2.96×10^{-3}

SUPPLEMENTARY FIGURE 10

ROPI rescues aberrant RNA splicing caused by the $TARDBP^{M337V/+}$ mutation. A. Scatterplot comparing the results of pairwise analysis between *TARDBP*$^{M337V/+}$ and *TARDBP*$^{+/+}$ at 84 h, and the analysis of *TARDBP*$^{M337V/+}$ under ROPI/DMSO at 84 h, after conversion to directionally adjusted *P* values. B. GO/KEGG pathway analysis for gene sets protective regulated by ROPI.

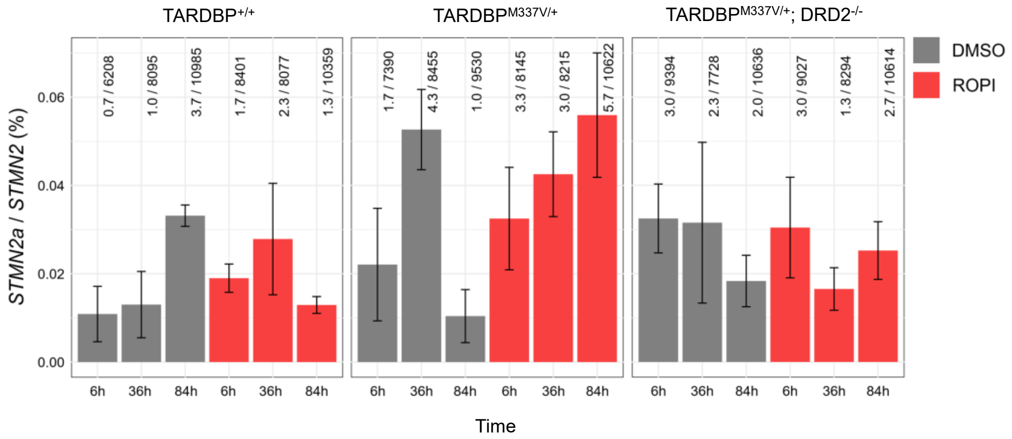

SUPPLEMENTARY FIGURE 11

Expression of the cryptic exon of STMN2 revealed by transcriptomic analysis.

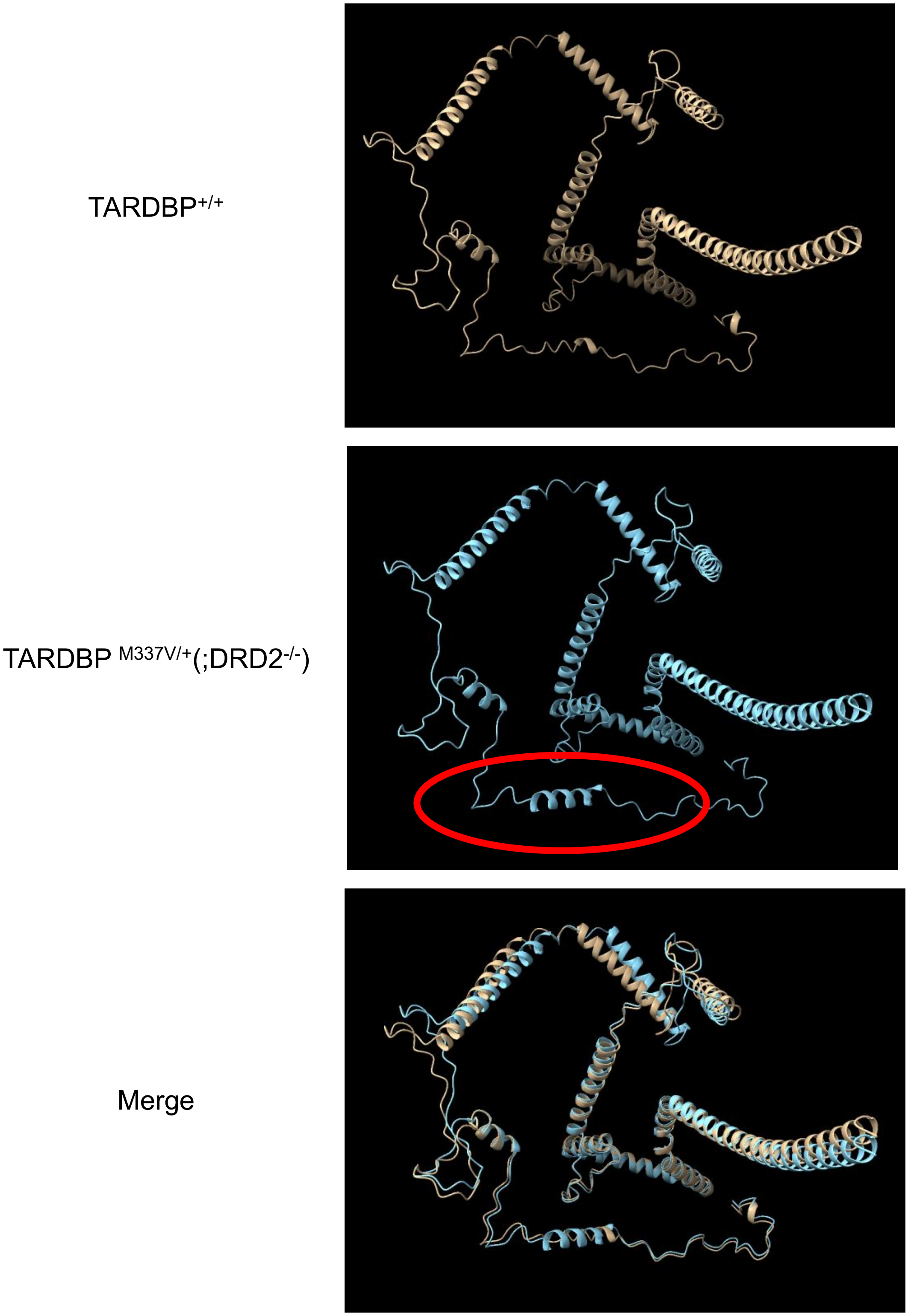

SUPPLEMENTARY FIGURE 12
Structure of the cryptic protein of DCTN2 by alpha fold database. The red circle highlights the novel alpha-helix structure.

PCR			
Gene	Forward	Reverse	application
GAPDH	CCACTCCTCCACCTTTGACG	ATGAGGTCCACCACCCTGTT	RT-qPCR
TUBB3	ATTTCATCTTTGGTCAGAGTGGGGC	TGCAGGCAGTCGCAGTTTTCAC	RT-qPCR
MAP2	GGATCAACGGAGAGCTGAC	TCAGGACTGCTACAGCCTCA	RT-qPCR
SYN1	TTGCCCAGATGGTTCGACTG	TGTCAACCTTGACCTTGCCC	RT-qPCR
HB9	GTCCACCGCGGGCATGATCC	TCTTCACCTGGGTCTCGGTGAGC	RT-qPCR
ChAT	GGAGGCGTGGAGCTCAGCGACACC	CGGGGAGCTCGCTGACGGAGTCTG	RT-qPCR
VACHT	GCTGTTTGCTTCCAAGGCTATCC	GAAGGCGAACAGGACTGTAGAG	RT-qPCR
DRD1	TGGTCTGTGCTGCCGTTATCAG	CAATCTCAGCCACTGCCTTCCA	RT-qPCR
DRD2	CAATACGCGCTACAGCTCCAAG	GGCAATGATGCACTCGTTCTGG	RT-qPCR
DRD3	TTGCTGTGTCCTGCCCTCTTCT	CTGGCATAGACAAGGACAGTCAC	RT-qPCR
DRD4	TGCTGCCGCTCTTCGTCTACTC	ACAGGTTGAAGATGGAGGCGGT	RT-qPCR
DRD5	CCGACGTGAATGCAGAGAACTG	TAGATGCGCGTGTAGGTCACGA	RT-qPCR
LARGE1	GAGCAGTGCTACAGAGACGTGT	TGCCGTCATACTCCAGGAAGGT	RT-qPCR
RAB27B	TGGCAACAAGGCAGACCTACCA	CTCCACATTCTGTCCAGTTGCTG	RT-qPCR
HOXB1	TGCCCTTCAGAACCTAACACCC	AGCTGCCTTGTGGTGAAGTTGG	RT-qPCR
VCAN	TTGGACCTCAGGCGCTTTCTAC	GGATGACCAATTACACTCAAATCAC	RT-qPCR
TSPYL5	GCATGGATACGCTGGAGAACGT	TTTGGATGAGGTGGTTCCTGCG	RT-qPCR
RIMBP2	AGGACGCCTATGACTCTCCAGA	CTCTGTGTGGTACTCGTCTCCA	RT-qPCR
NKTR	GCAAGCAGTTCAGAAGAGCCAAG	TCTCAGGCACTGGAGGAATCTC	RT-qPCR
LAT	ATCCTGGAGCGGCTAAGACTGA	GTTCAGCTCCTGCAGATTCTCG	RT-qPCR
RHBDL1	TGGCGCTTCCTCACCTACATGT	TGCCAGGTAGAGCAGGCTGATG	RT-qPCR
PILRB	TGGCACCTAAGTCTGGACACTG	CTCTGTTGGTCAGAAGTCACTGC	RT-qPCR
DRD2 FL mRNA	CAGTGGCTCCACCGCCCTG	TCAGCAGTGGAGGATCTTCAGGAA	RT-PCR
TARDBP	TTATAGATTGCGCAGTCTCTTTGTGG	CCCCAACCAATTGCTGCACCAG	Genome PCR
DRD2	ATTTGTCCTACCAGCTGTAAAAACTTGCTA	CTGGGAATTCCTTTAGCCTAGACC	Genome PCR
DRD2 Ex1	ATTTGTCCTACCAGCTGTAAAAACTTGCTA	GGAGAAAGTGCTGGAGCAAGCAG	Genome PCR
DRD2 Ex4	CTGGGAATTCCTTTAGCCTAGACC	TCTGGTATCTGCTGTGCGTTTGTG	Genome PCR

SUPPLEMENTARY TABLE 1 Nucleotide

Genome editing		
Name	Nucleotide	application
ssODN	GGTGGTGGGATGAACTTTGGTGCGTTCAGCATTAATCCAGCCATGATGGCTGCCGCCC AGGCAGCACTACAAAGTAGCTGGGGTATGGTCGGCATGTTAGCCAGCCAGCAGAACC AGTCAGGCCCATCGGGTAATAACCAAAACCAAGGCAACATGCAG	ssODN
crTARDBP	ACAGAGCAGUUGGGGUAUGA	crRNA
crDRD2-1	UGGAUCCACUGAAUCUGUCC	crRNA
crDRD2-2	GUUUGGUGUUGACUCGCUUG	crRNA

Plasmid		
REAGENT or RESOURCE	SOURCE	IDENTIFIER
pPB-Puro-TRE3G>hNEUROG2:T2A:hISL1:T2A:hLHX3	Vectorbuilder	VB190203-1017epw
pRP[Exp]-Puro-CMV>hyPBase	Vectorbuilder	VB180828-1458zzh
pPB-Hygro-EF1A>Tet3G	Vectorbuilder	VB180907-1018tew
pRP[CRISPR]-Bsd-hCas9-U6>{BbsI site}	Vectorbuilder	VB190123-1041kzp

SUPPLEMENTARY TABLE 1. (continued)

Antibody	Source	Cat No
βIII tubulin	Millipore	AB9354
HB9	DSHB	81.5C10
Dopamin D2 receptor	FRONTIER INSTITUTE	D2R-Rb-Af960
CREB	Cell Signaling Technology	9104
Phospho-CREB (Ser133)	Cell Signaling Technology	9198
β-actin	Sigma-Aldrich	A1978

SUPPLEMENTARY TABLE 2. Antibody

REAGENT or RESOURCE	SOURCE	IDENTIFIER
Biological samples, chemicals and recombinant proteins		
StemFit AK02N	Ajinomoto	AK-02N
Y-27632	Chemscene	CS-0878
iMatrix-511 silk	Takara Bio	T311
Penicillin-Streptomycin	Nacalai Tesque	18188-04
TrypLE Select	ThermoFisher Scientific	12563-029
Opti-MEM	ThermoFisher Scientific	31985062
Lipofectamine Stem Transfection Reagent	ThermoFisher Scientific	STEM00001
Puromycin	InvivoGen	ant-pr-1
Blasticidin	InvivoGen	ant-bl-05
Hygromycin B Gold	InvivoGen	ant-hg-1
StemCellBanker	ZENOAQ	11924
poly-D-lysine	Sigma-Aldrich	P0899
Matrigel	Corning	BD354277
KBM Neural Stem Cell	Kohjin Bio	16050100
B-27 Supplement	ThermoFisher Scientific	17504001
B-27 Supplement minus AO	ThermoFisher Scientific	10889038
CHIR99021	Sigma-Aldrich	SML1046
LDN-193189	StemRD	SRD LDN-010
SB431542	Tocris Bioscience	1614/10
DAPT	Selleck	S2215
Recombinant human BDNF protein (Animal Free)	Abcam	ab222178
Recombinant human GDNF protein	Alomone Labs	G-240
PD0332991	Selleck	S1116
Retinoic acid	Sigma-Aldrich	R2625
Ascorbic acid	Sigma-Aldrich	A4544
Doxycycline hyclate	Sigma-Aldrich	D9891
Neruobasal plus medium	ThermoFisher Scientific	A3582901
B-27 Plus supplement	ThermoFisher Scientific	A3582801
Culture One supplement	ThermoFisher Scientific	A3320201
dbcAMP	Selleck	S7858
NT-3	Alomone labs	N-260
purmorphamine	Sigma-Aldrich	483367-10-8
Ropinirole	Selleck	S3189
5-Hydroxyoxindole	Combi-Blocks	IN-0505
Bovine Serum Albumin	Sigma-Aldrich	A9647-10G
Triton X-100	Sigma-Aldrich	X100-100ML
RIPA	Nacalai Tesque	16488-34
protease inhibitor cocktail	Nacalai Tesque	04080-11
Phosphatase Inhibitor Cocktail	Nacalai Tesque	07575-51
polyethyleneimine	Sigma-Aldrich	P3143
Probe and Kit		
SYTOX Green	ThermoFisher Scientific	S34860
CellROX Green	ThermoFisher Scientific	C10444
Tubulin Tracker Deep Red	ThermoFisher Scientific	T34077
RNeasy Plus Mini Kit	QIAGEN	74136
ReverTra Ace qPCR RT Kit	TOYOBO	FSQ-101
THUNDERBIRD SYBR qPCR Mix	TOYOBO	QPS-201
Micro BCA Protein Assay	ThermoFisher Scientific	23235
ECL Prime Western Blotting System	Amersham	RPN2232
12-230 kDa Jess or Wes Separation Module	ProteinSimple	SM-W004
iPSC		
RPChiPS8023G1	REPROCELL	RCRP004N

SUPPLEMENTARY TABLE 3. Reagent or resource

Quantitative analysis of CREB phosphorylation

Comparison	Test	df	Test statistic	P-value
TARDBP$^{M337V/+}$ (DMSO vs ROPI)	Welch's t-test	df = 2	t = 50.23	0.00040
TARDBP$^{M337V/+}$; DRD2$^{-/-}$ (DMSO vs ROPI)	Welch's t-test	df = 2	t = -1.44	0.29

Cell death assays

Comparison	Test	df	Test statistic	P-value
Bartlett test (all groups)	Bartlett test	df = 4	0.67402	0.95
ANOVA (all groups)	ANOVA	df = 4	F = 69.13	0.000000302
TARDBP$^{+/+}$ vs TARDBP$^{M337V/+}$	Tukey's test	*	*	0.000
TARDBP$^{M337V/+}$ +ROPI vs TARDBP$^{M337V/+}$	Tukey's test	*	*	0.038
TARDBP$^{+/+}$ vs TARDBP$^{M337V/+}$:DRD2$^{-/-}$	Tukey's test	*	*	0.000
TARDBP$^{M337V/+}$:DRD2$^{-/-}$ +ROPI vs TARDBP$^{M337V/+}$:DRD2$^{-/-}$	Tukey's test	*	*	0.014
TARDBP$^{M337V/+}$ vs TARDBP$^{M337V/+}$:DRD2-/-	Tukey's test	*	*	0.023
TARDBP$^{M337V/+}$ +ROPI vs TARDBP$^{M337V/+}$:DRD2$^{-/-}$ +ROPI	Tukey's test	*	*	0.063

ROS production assay

Comparison	Test	df (or num, denom)	Test statistic	P-value
Bartlett test (TARDBP$^{+/+}$, TARDBP$^{M337V/+,}$ TARDBP$^{M337V/+}$ +ROPI)	Bartlett test	df = 2	10.462	0.005349
Welch ANOVA (TARDBP+/+, TARDBPM337V/+, TARDBPM337V/+ +ROPI)	Welch ANOVA	df = (2, 12.37)	F = 82.96	6.8E-08
TARDBP$^{+/+}$ vs TARDBP$^{M337V/+}$	games_howell_test	**	**	0.000
TARDBP$^{M337V/+}$ +ROPI vs TARDBP$^{M337V/+}$	games_howell_test	**	**	0.027
Bartlett test (TARDBP$^{+/+}$, TARDBP$^{M337V/+}$; DRD2$^{-/-,}$ TARDBP$^{M337V/+}$; DRD2$^{-/-}$ +ROPI)	Bartlett test	df = 2	1.3948	0.4979
Welch ANOVA (TARDBP$^{+/+}$, TARDBP$^{M337V/+}$; DRD2$^{-/-,}$ TARDBP$^{M337V/+}$; DRD2$^{-/-}$ +ROPI)	Welch ANOVA	df = (2, 5.37)	F = 114.96	3.9E-05
TARDBP$^{+/+}$ vs TARDBP$^{M337V/+}$; DRD2$^{-/-}$	games_howell_test	**	**	0.000
TARDBP$^{M337V/+}$; DRD2$^{-/-}$ +ROPI vs TARDBP$^{M337V/+}$; DRD2$^{-/-}$	games_howell_test	**	**	0.003

MEA

Comparison	Test	df (or num, denom)	Test statistic	P-value
Bartlett test (all groups)	Bartlett test	df = 2	K^2 = 1194.6	3.9E-260
Welch ANOVA(all groups)	Welch ANOVA	df = (2, 24.66)	F = 11.423	3.1E-04
TARDBP$^{+/+}$ vs TARDBP$^{M337V/+}$	games_howell_test	**	**	0.002
TARDBP$^{+/+}$ vs TARDBP$^{M337V/+}$:DRD2$^{-/-}$	games_howell_test	**	**	0.038
TARDBP$^{M337V/+}$ vs TARDBP$^{M337V/+}$:DRD2-/-	games_howell_test	**	**	0.60

MEA (ROPI vs DMSO)

Comparison	Test	df (or num, denom)	Test statistic	P-value
F-test (TARDBPM337V/+ (DMSO vs ROPI))	F test	df = (16, 21)	F = 0.00031	2.20E-16
TARDBP$^{M337V/+}$ (DMSO vs ROPI)	Welch's t-test	df = 21.017	t = -3.46	0.0023
F-test (TARDBPM337V/+; DRD2-/- (DMSO vs ROPI))	F test	df = (18, 17)	F = 11.77	0.0000052
TARDBP$^{M337V/+}$; DRD2$^{-/-}$ (DMSO vs ROPI)	Welch's t-test	df = 21.19	t = 2.23	0.037

MEA: ROPI-Induced Frequency Reduction

Comparison	Test	df (num, denom)	Test statistic	P-value
F-test (TARDBPM337V/+ (DMSO vs ROPI))	F test	df = (17,16)	F = 220.74	1.78E-15
TARDBP$^{M337V/+}$ (DMSO vs ROPI)	Welch's t-test	df = 17.16	t = -4.29	0.00049

* Test statistic and degrees of freedom are not provided by the Tukey's test in EZR output.

** Test statistic and degrees of freedom not reported by the Games-Howell method.

SUPPLEMENTARY TABLE 4

Detailed statistical analysis of all experiments.

Statistical tests used for each assay are indicated along with degrees of freedom, test statistics, and p-values. Welch's t-test, ANOVA with Tukey's post hoc test, Welch ANOVA with Games-Howell test, and F-tests were applied as appropriate. See Methods for details.

	Locus	Position	CFD Score	Mutation
TARDBP gRNA	intergenic RP11-317M22.1-SLC22A2	chr6:160170427 (-)	0.595	No
	intron GGH	chr8:63034809 (-)	0.59	No
	intron RAB37	chr17:74739046 (-)	0.453	No
	exon RP11-16E18.1	chr8:63136638 (-)	0.448	No
	intron OVCH1	chr12:29428591 (-)	0.402	No
	intron BMPR1B	chr4:95051892 (-)	0.375	No
	intron PACS1	chr11:66140635 (-)	0.366	No
	intron CCNY	chr10:35342829 (-)	0.338	No
	intergenic BRINP2-RP1-35C21.1	chr1:177337457 (-)	0.338	No
	intron ENTPD4	chr8:23400614 (-)	0.241	No
DRD2 gRNA #1	intron DGKB	chr7:14349938 (-)	0.509	No
	intergenic RP11-347C18.3-NDUFAF6	chr8:94951967 (-)	0.287	No
	intergenic RP11-118G23.1-AC024610.1	chr17:33761457 (-)	0.247	No
	intergenic RP11-68E19.1-AC017002.4	chr2:111585890 (-)	0.246	No
	intron SLC38A5	chrX:48467682 (-)	0.218	No
	intergenic CSNK2A1-TCF15	chr20:591286 (-)	0.218	No
	intron IGDCC4	chr15:65393675 (-)	0.205	No
	intergenic RP11-82L7.4-ANXA2	chr15:60138980 (-)	0.183	No
	intergenic RNU6-184P-SURF6P1	chrY:17173547 (-)	0.17	Chromosome Y
	intron CTD-2378E21.1	chr17:70326583 (-)	0.159	No
DRD2 gRNA #2	intergenic AC026202.5-AC027119.1	chr3:5550179 (-)	0.583	No
	intergenic FBXL7-RNA5SP178	chr5:15969149 (-)	0.282	No
	exon AP1G2/RP11-66N24.3/JPH4	chr14:23568771 (-)	0.258	No
	intron BCAR3	chr1:93680737 (-)	0.128	No
	intergenic AC092038.1-AC092038.2	chr3:23414469 (-)	0.115	No
	intergenic LSAMP-RNU6-1200P	chr3:117359570 (-)	0.102	No
	intergenic RP11-558O12.1-RP11-706O15.1	chrX:3811776 (-)	0.097	No
	intergenic RP11-10E6.1-EIF3IP1	chr7:109765097 (-)	0.096	No
	exon RPL13A/SNORD32A/SNORD33/SNORD34/SNORD35A	chr19:49490494 (-)	0.072	No
	exon RP11-564A8.4	chr1:206907792 (-)	0.072	No
	exon RPL13AP5	chr10:96750461 (-)	0.072	No

SUPPLEMENTARY TABLE 5. List of potential off-targets by UCSC Database

Ferroptosis-Suppressor Genes

Gene symbol	*TARDBP*$^{+/+}$	*TARDBP*$^{M337V/+}$	*TARDBP*$^{M337V/+}$; *DRD2*$^{-/-}$	*TARDBP*$^{+/+}$ +ROPI	*TARDBP*$^{M337V/+}$ +ROPI	*TARDBP*$^{M337V/+}$; *DRD2*$^{-/-}$ +ROPI
GPX1	1.00	0.66	0.62	1.04	1.08	0.94
GPX4	1.00	0.76	0.73	1.02	0.99	0.96
FTL	1.00	0.76	0.68	1.08	1.30	1.16
FTH1	1.00	0.80	0.82	0.99	1.21	1.14

Ferroptosis-Promoting Genes

Gene symbol	*TARDBP*$^{+/+}$	*TARDBP*$^{M337V/+}$	*TARDBP*$^{M337V/+}$; *DRD2*$^{-/-}$	*TARDBP*$^{+/+}$ +ROPI	*TARDBP*$^{M337V/+}$ +ROPI	*TARDBP*$^{M337V/+}$; *DRD2*$^{-/-}$ +ROPI
TFRC	1.00	2.28	2.51	0.93	1.09	1.24
DMT1	1.00	1.97	1.89	1.02	1.31	1.31

SUPPLEMENTARY TABLE 6.
Heatmap visualization of ferroptosis-related gene expression revealed by transcriptomic analysis.

Gene Symbol	*TARDBP*$^{+/+}$	*TARDBP*$^{M337V/+}$	*TARDBP*$^{M337V/+}$; *DRD2*$^{-/-}$	*TARDBP*$^{+/+}$ +ROPI	*TARDBP*$^{M337V/+}$ +ROPI	*TARDBP*$^{M337V/+}$; *DRD2*$^{-/-}$ +ROPI
SCN1B	1	1.04	1.03	0.80	0.46	0.46
SCN2A	1	1.14	1.47	1.00	0.63	0.82
SCN2B	1	1.72	2.27	1.16	1.53	1.81
SCN3A	1	1.33	1.41	0.95	0.77	0.82
SCN3B	1	0.96	1.13	0.99	0.67	0.84
SCN5A	1	2.85	1.67	1.31	1.20	1.61
SCN7A	1	1.42	1.15	0.84	0.81	0.72
SCN8A	1	2.52	2.89	0.95	0.81	1.05
SCN9A	1	1.27	1.52	0.97	0.56	0.72

SUPPLEMENTARY TABLE 7.
Heatmap visualization of voltage-gated sodium channel gene expression revealed by transcriptomic analysis.
